# Warmer Lakes Support Phytoplankton Over Fish

**DOI:** 10.1111/gcb.70288

**Published:** 2025-06-09

**Authors:** Benjamin Paul Mooney, Anna Gårdmark, Carolyn Faithfull, Renee Mina van Dorst, Magnus Huss

**Affiliations:** ^1^ Department of Aquatic Resources Swedish University of Agricultural Sciences Uppsala Sweden; ^2^ Department of Wildlife, Fish and Environmental Studies Swedish University of Agricultural Sciences Umeå Sweden

**Keywords:** aquatic food webs, biomass distribution, climate warming, energy transfer efficiency, temperature gradient

## Abstract

Climate warming reshapes biomass distributions across trophic levels in aquatic systems, with implications for ecosystem functioning and service provisioning. Using a space‐for‐time approach across temperate and boreal lakes, we analyse a dataset spanning wide gradients in temperature and nutrient availability, including species and biomass data for phytoplankton, fish, and, in some cases, zooplankton. We hypothesise that (1) warmer lakes have higher fish‐to‐phytoplankton biomass ratios than colder lakes, and (2) this relationship weakens at high phosphorus levels due to proliferation of inedible phytoplankton. Contrary to expectations, our results show that warmer lakes exhibit lower fish‐to‐phytoplankton biomass ratios, regardless of phosphorus concentrations or the contribution of benthic relative to whole lake primary production. This suggests reduced energy transfer efficiency from producers to consumers in warming waters. Changes in phytoplankton and fish community composition are likely part of the explanation for why increased phytoplankton biomass in warmer lakes does not translate into higher fish biomass. Our findings highlight a critical shift in biomass distribution from fish to phytoplankton with rising temperatures in northern lakes, potentially signalling future declines in food web efficiency and predator biomass under continued climate warming.

## Graphical abstract

Climate warming is changing the balance of food webs in lakes. We studied northern lakes and found that warmer waters tend to support more phytoplankton but not more fish, suggesting that less energy is making its way up the food chain. This shift in biomass may affect the services lakes provide, such as fisheries.

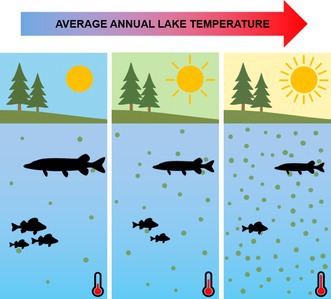

## 1 Introduction

Rising global surface temperatures (Blunden and Arndt 2016; IPCC 2023) have profound consequences for lakes, including the loss of ice cover, changes in evaporation and water budgets, warming of surface waters, and alterations in mixing regimes (Woolway et al. 2020). Predicting the consequential effects of warming on lake food webs is challenging due to different responses among organism groups (O'Connor et al. 2009; Shimoda et al. 2011; Sommer et al. 2012) and simultaneous changes in other environmental drivers, such as precipitation patterns (IPCC 2023) and nutrient concentrations (Glibert and Burford 2017; Isles et al. 2018). Furthermore, these changes occur at different rates across geographical gradients. Northern Europe, for instance, is experiencing an increase in annual precipitation (IPCC 2023) and warming at a rate exceeding the global mean (Pörtner et al. 2022). Aquatic ecosystem responses to such changes can include shifts in the relative importance of bottom‐up vs. top‐down control (Shurin et al. 2012; Tanentzap et al. 2020), biomass re‐distributions across trophic levels (Bideault et al. 2021) and shifts in species composition (Anderson et al. 2022; Havens et al. 2009; Tavşanoğlu et al. 2017). Such responses may threaten ecosystem functioning and stability by triggering shifts in ecological interactions and trophic cascades (Bonnaffé et al. 2024; Mei et al. 2022; Möllmann et al. 2008), resulting from and also modifying the pathways and efficiency of energy flowing through food webs (e.g., Ullah et al. 2018).

Although gross primary production generally increases with temperature due to enhanced photosynthetic rates (O'Connor et al. 2009; Richardson and Schoeman 2004), the total biomass of primary producers often declines because respiratory rates also increase (Atwood et al. 2015; Bernhardt et al. 2018; Kwiatkowski et al. 2019). However, recent research suggests more complex temperature—phytoplankton relationships when accounting for concurrent environmental variation, such as nutrient concentration. For instance, warming can shift plankton communities toward smaller or less edible species such as cyanobacteria (Erratt et al. 2023), reducing energy transfer efficiency and altering biomass production of higher trophic levels (Tanentzap et al. 2020; Ullah et al. 2018). Such shifts in energy transfer, combined with changes in primary production (O'Connor et al. 2009) and prey community composition (Tavşanoğlu et al. 2017), are likely to influence aquatic consumers, including zooplankton and fish, through bottom‐up processes. Simultaneously, consumers are also subject to top‐down effects from higher trophic levels (Hessen and Kaartvedt 2014), including warming‐induced physiological changes in consumers, such as elevated metabolism (Brown et al. 2004; Kern et al. 2015) and altered feeding rates (Englund et al. 2011; Lindmark, Ohlberget, et al. 2022). Therefore, the relative changes in primary producer versus consumer biomass depend on the temperature sensitivity of phytoplankton carrying capacity, production, and species composition, as well as consumer metabolic rates and feeding rates (Fussmann et al. 2014; Gårdmark and Huss 2020).

For fish, both theoretical (Audzijonyte et al. 2023; Heneghan et al. 2023) and empirical evidence (Atkinson et al. 2024) suggest that fish community biomass generally declines under warming in response to decreasing phytoplankton production or biomass. However, responses may vary regionally, as some studies indicate that warming can amplify phytoplankton biomass, potentially due to increased cyanobacterial production (Erratt et al. 2023; Paerl and Huisman 2008). Nevertheless, many observations indicate differential responses between primary producers and consumers in response to warming (O'Connor et al. 2009; Yvon‐Durocher et al. 2015), often increasing the ratio of consumer‐to‐producer biomass (Jennings and Collingridge 2015). Empirical observations to this end have mainly been made under experimental warming (Müren et al. 2005; O'Connor et al. 2009) and have commonly found shifts towards top‐heavy food webs (Shurin et al. 2012), although there are also examples of experimental warming causing bottom‐heavy food webs (Nagelkerken et al. 2020).

Understanding when and how climate change alters the ratios of primary producer, consumer, and predator standing stock biomass and production is crucial, as these changes can affect species diversity (Zhang et al. 2017), interactions within (Gårdmark and Huss 2020) and between (O'Connor et al. 2009) species, and therefore overall ecosystem productivity and stability (Kovalenko 2019). However, many studies concerning aquatic food webs have focused narrowly on specific aspects, such as the effects of warming on individual trophic levels (e.g., primary producers or consumers) in isolation (O'Gorman et al. 2012) or on the dynamics of single predator–prey species (Boukal et al. 2019; Sentis et al. 2015). The same is true for the many studies on how nutrient availability relates to biomass distributions (Hessen et al. 2006; Jeppesen et al. 2005), and the much fewer studies on the interactive effects with temperature (Deng et al. 2014; Yang et al. 2019; Yin et al. 2023). Specifically, few studies have considered the relative change in biomass across multiple trophic levels under the interactive effects of temperature and nutrients (but see Bouraï et al. 2020). We therefore lack an understanding of how large‐scale temperature‐biomass relationships hold across trophic levels, especially when accounting for variation in nutrient availability.

In this study, we investigate how warmer waters affect the relative biomasses of trophic levels by examining phytoplankton, zooplankton, and fish biomasses in lakes along a natural growth season temperature gradient from 9°C to 18°C. We also test the extent to which these temperature‐driven biomass responses hold across gradients in phosphorus concentrations and in the proportion of benthic to whole lake primary production. Using long‐term monitoring data from up to 125 Swedish temperate and boreal lakes, we evaluate phytoplankton and fish data with a space‐for‐time approach. For a sub‐set of lakes, we also use data from annual sampling of phytoplankton, zooplankton, and fish. We specifically test two hypotheses: (1) warmer lakes have a higher fish relative to phytoplankton biomass than cold lakes, and (2) this relationship is weakened at higher phosphorus concentrations due to the proliferation of non‐edible resources.

## 2 Methods

### 2.1 Data and Lake Selection

**FIGURE 1:**
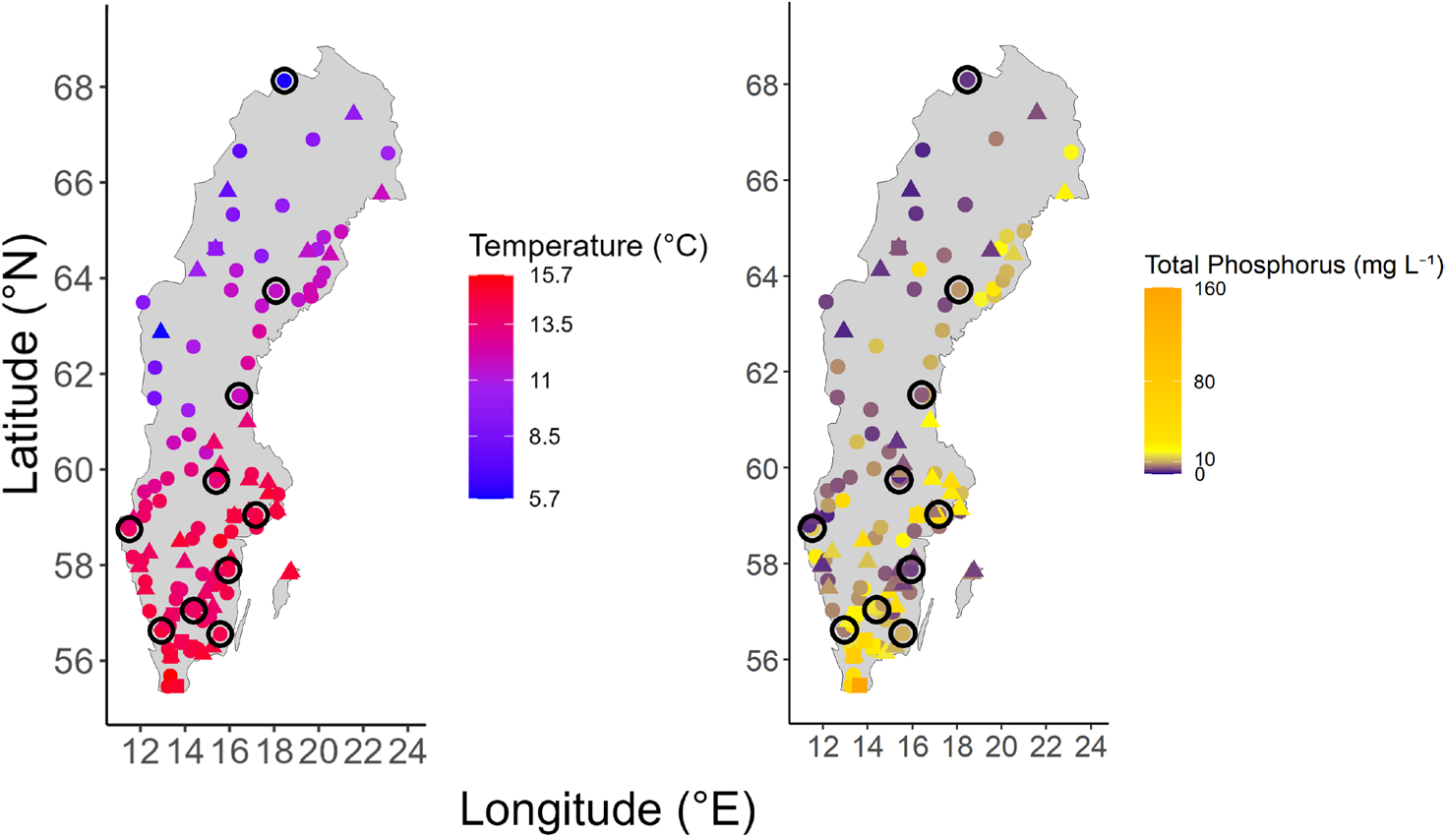
A map of Sweden marking the distribution of the 125 study lakes included in this analysis. Lakes are coloured based on the (left map) average air temperature and (right) average annual total phosphorus concentration of the lake recorded from May to September 2004–2023. Circles indicate lakes with both phytoplankton and fish data, triangles indicate lakes with only phytoplankton data, and squares indicate lakes with only fish data. Black rings mark the location of the 10 trend lakes.

We selected lakes across Sweden based on the following five criteria: (1) Lakes had an area between 0.1 and 10 km^2^, with larger lakes excluded from the analyses to limit variation due to lake size; (2) For analyses including phytoplankton, lakes had measurements of biovolume per phytoplankton family, and for analyses including fish, they had measurements of catch‐per‐unit‐effort (CPUE) biomass per fish species; (3) Lakes had measurements of total phosphorus concentration, absorbance at 420 nm, and information on lake area and maximum depth; (4) Lakes had been sampled in July and/or August for at least 2 years within the past 20 years (2004–2023). We chose July and August as most sampling occasions for lake monitoring concerning fish and phytoplankton are conducted in these months, and because late summer is appropriate for latitudinal comparisons concerning plankton in our study region (Suikkanen et al. 2013). (5) For analyses including both phytoplankton and fish data, the mean year of phytoplankton sampling and the mean year of fish sampling should differ by no more than 5 years, based on the premise that phytoplankton sampled beyond 5 years prior to fish would have little to no impact on fish biomass. These selection criteria resulted in a dataset of up to 125 (depending on the analysis) small to intermediately sized lakes (0.5–7.3 km^2^), distributed from north to south Sweden (68.29645°N—55.48314°N), covering a large range of productive season (May–September) air temperatures (9.08°C–17.87°C) and total phosphorus concentrations (2.04–62.87 μg L^−1^ total phosphorous) (Figure 1, Table S1).

### 2.2 Water Temperature

We chose to represent the annual thermal environment for the different lakes using continuous growth season air temperature estimates instead of the sparsely sampled water temperatures (correlation *r* = 0.883; 95% CI: 0.907–0.961; *p* < 0.05), as this is likely to give a closer representation of the thermal climate of each lake than would single water samples taken in July and/or August (Sharma et al. 2008). The average growth season air temperature was calculated as the yearly mean of the average air temperatures from May to September for the past 20 years (2004–2023), using hourly estimates of temperature data per 4 km^2^ land mass from SMHI's Precipitation Temperature Hydrological Agency's Water Model (Swedish Meteorological and Hydrological Institute 2024) (PTHBV, https://www.smhi.se/data/nederbord‐och‐fuktighet/nederbord/griddad‐nederbord‐‐och‐temperaturdata, on 2024‐04‐01).

### 2.3 Phytoplankton, Zooplankton, and Water Geochemistry

We obtained phytoplankton, zooplankton, and water geochemical data from the Miljödata MVM database (MVM, https://miljodata.slu.se/MVM/, on 2024‐04‐01). Water samples were taken at a depth of 0.5 m and were all analyzed at the Department of Aquatic Sciences and Assessment, Swedish University of Agricultural Sciences 2024. The analytical procedures followed international (ISO) or European (EN) standards (SS‐EN ISO 5667‐1:2007). Using these data, we estimated mean total phosphorous concentration (TP) per lake over the entire 20‐year period, which was used as a proxy for nutrient availability. We acknowledge that both phosphorous and nitrogen can limit primary production in our study region (Bergström et al. 2008; Elser et al. 2009). However, phosphorus and nitrogen concentrations are strongly correlated in the data used for this study (*r* = 0.800; 95% CI: 0.709–0.864; *p* < 0.05). To avoid multicollinearity, we therefore chose to only include phosphorous as a covariate, which for the majority of our study lakes is likely to be the most limiting nutrient considering most are located in southern Sweden (Liess et al. 2009). Phytoplankton were collected from the epilimnion using one to several 2‐m long tubes, depending on the size of the lake. Samples were mixed to form a composite sample, from which subsamples were taken for analyses. Phytoplankton were preserved with acid Lugol's iodine solution and stored at 4°C–5°C in the dark prior to analysis. Phytoplankton were counted using an inverted light microscope and a modified Utermöhl (1958) technique according to Olrik et al. (1998). Biovolume (μg L^−1^) was estimated using geometric forms and biomass from counts of individuals of each taxon (Olrik et al. 1998). Zooplankton samples were collected using a 4.2‐L Limnos water sampler at 2 m depth intervals, from which individuals were collected using a 40‐μm net and preserved with alkaline Lugol's iodine solution. Phytoplankton and zooplankton were identified to the lowest possible taxonomic level by microscopy by taxonomists at the Department of Aquatic Sciences and Assessment, Swedish University of Agricultural Sciences (see Tables S11 and S12 for the most used taxonomic resources).

### 2.4 Fish

Fish data was obtained from the Swedish National Register of Survey test fishing (Swedish University of Agricultural Sciences, Department of Aquatic Resources, D. of A. R. 2024) (NORS, https://www.slu.se/sjoprovfiskedatabasen/, on 2024‐04‐01). Fish were sampled according to European standards. In the benthic zone, benthic NORDIC multi‐mesh gillnets were used (45 m^2^; 30 m long × 1.5 m high), consisting of 12 panels, each 2.5 m wide, with mesh sizes ranging from 5 to 55 mm (Appelberg et al. 1995). In the pelagic zone, fish were sampled using floating multi‐mesh gillnets (165 m^2^; 27.5 m long × 6 m high), consisting of 11 panels, each 2.5 m wide, with mesh sizes ranging from 6.25 to 55 mm (Appelberg 2000). The total number of benthic gillnets used when sampling is standardized based on the size and depth of the lake and is set randomly over the entire lake within fixed depth strata (0–2.9, 3–5.9, 6–11.9, 12–19.9, 20–34.9, 35–49.5, 50–74.9, and > 75 m). Gillnets were set from 19:00 to 07:00 the following day to include fishing during dusk and dawn. In the main analyses of this study, only fish sampled with benthic gillnets are used. This is because pelagic nets are only used in a subset of lakes deeper than 10 m (only 49 of the selected lakes were deep enough for pelagic nets to be set), whereas benthic nets are used in all study lakes (CEN 2015). Moreover, whereas benthic net sampling is scaled by lake size, the number of pelagic nets is not and is therefore less useful for relating fish catches to other variables at a whole lake scale. Even when pelagic net sampling occurs, benthic nets on average represent 65% of the CPUE of fish caught in our study lakes. Still, we performed supplementary analyses that also include the pelagic nets, which produced similar results in terms of significance as in the main analysis using benthic nets only (Table S9).

The catch in the benthic nets is representative of most fish species in this type of lakes (Appelberg et al. 1995). However, northern pike ( *Esox lucius* ) and European eel ( *Anguilla anguilla* ) are not representatively caught in gillnet fishing due to their behavior and shape, and since these fish made up just 0.22% of the total abundance, they were removed from the analysis. In our analyses, we used catch‐per‐unit‐effort of the whole‐community biomass of all other species.

### 2.5 Benthic Proportion of Primary Production and Lake Morphometry

We estimated habitat‐specific gross primary production by benthic algae (i.e., epilithic) and phytoplankton (i.e., pelagic), from which we calculated the ratio of benthic production to whole lake primary production (“B/P production ratio”) as per Norman et al. (2022; Table 2). Estimates of the B/P production ratio require measured lake morphometry, light attenuation in the water column, and predefined values for maximum productivity of benthic algae and phytoplankton. The predefined values for benthic production were obtained from measured production in soft sediments (50 C m^−2^ H^−1^; Vadeboncoeur et al. 2008; 25 and 100 C m^−2^ H^−1^ were used in sensitivity analyses, but the outcome of the statistical analyses did not qualitatively change) and rocky shores (5 C m^−2^ h^−1^; Vadeboncoeur et al. 2008), and the average pelagic production from measurements in four Swedish lakes (1.9 C m^−2^ H^−1^; Heyman 1983). The light extinction coefficient was calculated using absorbance values, according to Seekell et al. (2015), from water sampled concurrently with phytoplankton and nutrients. Global irradiance data, needed to calculate light availability was obtained from SMHI's STRÅNG model (https://opendata.smhi.se/apidocs/strang/, on 01/04/2024) and was averaged per year. Lake size and depth data was obtained from the NORS database (NORS, 2024).

The B/P production ratio was included as a covariate in our analyses for three reasons: (1) Because we expect the benthic and pelagic habitats to respond differently to TP since pelagic but not benthic primary production is often nutrient limited (e.g., Krause‐Jensen et al. 2012). (2) Because the pathway from primary production to higher trophic levels differs between benthic and pelagic habitats, which may also imply differences in how efficiently energy flows from producers to consumers. (3) The biomass of pelagic primary producers but not benthic primary producers is included as a response variable in this study due to data limitation, and the B/P production ratio therefore acts as a control for this lack of benthic data in the analysis.

The biomass (or biovolume) of all trophic levels was converted to carbon mass but will from hereon be referred to as biomass. The conversion to carbon mass negates differences in biochemical compositions between organisms and allows for comparisons between trophic levels using a common standardised unit. Phytoplankton biovolume was converted to carbon mass using a conversion factor of 0.11, except for cyanobacteria and chlorophytes where conversion factors of 0.22 and 0.16 were used, respectively (Blomqvist et al. 1995; Table S4). Zooplankton biovolume was converted to carbon mass using a conversion factor of 0.1 (Postel et al. 2000). The mean carbon mass of phytoplankton, mean carbon mass of zooplankton, and mean concentration of TP were all calculated as an average per litre, per lake, across the entire time period. Fish biomass values were converted to carbon mass per unit effort using a conversion factor of 0.1 (Czamanski et al. 2011).

### 2.6 Statistical Analysis

To test whether (i) warmer lakes have biomass distributions characterised by less phytoplankton relative to predators (fish), and whether (ii) this difference is reduced at higher nutrient concentrations (TP), we used a single multiple linear regression model with interaction terms. Specifically, we included temperature as the main predictor, with an interaction term for TP to evaluate its modifying effect on temperature‐driven biomasses and biomass distributions. TP was included to account for large variation in nutrient availability between lakes. We also included the benthic to whole lake primary production (B/P production ratio) as a covariate to account for (1) differing TP responses in benthic vs. pelagic systems, (2) potential variation in energy transfer efficiencies between habitats, and (3) the exclusion of benthic primary producers from our response variables. By focusing on interactions rather than main effects, we directly test how the effect of temperature is modulated by TP and further shaped by the B/P production ratio.

Response variable ~ temperature + temperature × TP + temperature × TP × B/P production ratio.

The response variables were ln‐transformed to obtain a normal distribution of the model residuals. Prior to analysis, we used the Variance Inflation Factor (VIF; Fox and Weisberg 2019) to test for multicollinearity between explanatory variables (all VIF = 1). Model residuals were checked for heteroscedasticity using Breusch‐Pagan tests (Zeileis and Hothorn 2002) and the normality of residuals was checked visually and using Shapiro–Wilk tests (Figures S4 and S5). For the main analysis, outliers were identified based on Cook's distance, and a few lakes were accordingly removed. The removal of these lakes did not affect the result of the analysis (results not shown) but was necessary to meet the assumption of normally distributed model residuals.

### 2.7 Trend Lake Analysis

In an attempt to resolve where energy losses are occurring in between phytoplankton and fish trophic levels depending on temperature, we ran mixed‐effects linear regression analyses (Pinheiro and Bates 2000) on a subset of 10 lakes where intermediate trophic levels (zooplankton) have been measured (Figure 3, Table 2). These so‐called trend lakes (https://www.slu.se/institutioner/vatten‐miljo/miljoanalys/sjoar‐och‐vattendrag/trendsjoar/) have been sampled multiple times per year since 2007 for water chemistry, phytoplankton, zooplankton, and fish. Water chemistry is sampled monthly during the ice‐free season, phytoplankton and zooplankton are sampled four times per year, and test fishing is conducted annually. Using this data, we analyzed the response of biomass ratios to variation in temperature over time. The response variables included: (1) the biomass ratio of fish to phytoplankton (F/P ratio), (2) the biomass ratio of zooplankton to phytoplankton (Z/P biomass ratio), and (3) the biomass ratio of fish to zooplankton (F/Z biomass ratio). In these models, yearly mean growth season temperature was treated as a fixed effect, while a random intercept and slope model was applied to account for the hierarchical structure of the data, with year nested within lake (random = ~1 + year | lake). This nesting structure recognizes that repeated measures from the same lake across years are not independent.

To explore whether similar temperature‐driven trends were evident also in the individual trophic groups, we tested how the biomass of phytoplankton, zooplankton, and fish as separate response variables varied with temperature over time. This allowed us to assess whether temporal changes in biomass within lakes were consistent with the trends observed in the large‐scale spatial analyses. We included only temperature in these temporal models because we have good annual estimates for temperature, but only single samples per year for nutrient concentration and little variation in B/P production ratio between years. In these models, fish biomass in year *t* + 1 is compared with phytoplankton or zooplankton biomass in year *t* (multiple lag times were tested and the result of the linear models were not qualitatively different; Tables S2 and S3). This is because we do not expect fish community production and biomass to respond to variation in resource availability within the same season. All data handling and statistics were done using R version 4.2.2 (R Core Team 2022) and visualized using the package *ggplot2* (Wickham 2016).

## 3 Results

**FIGURE 2:**
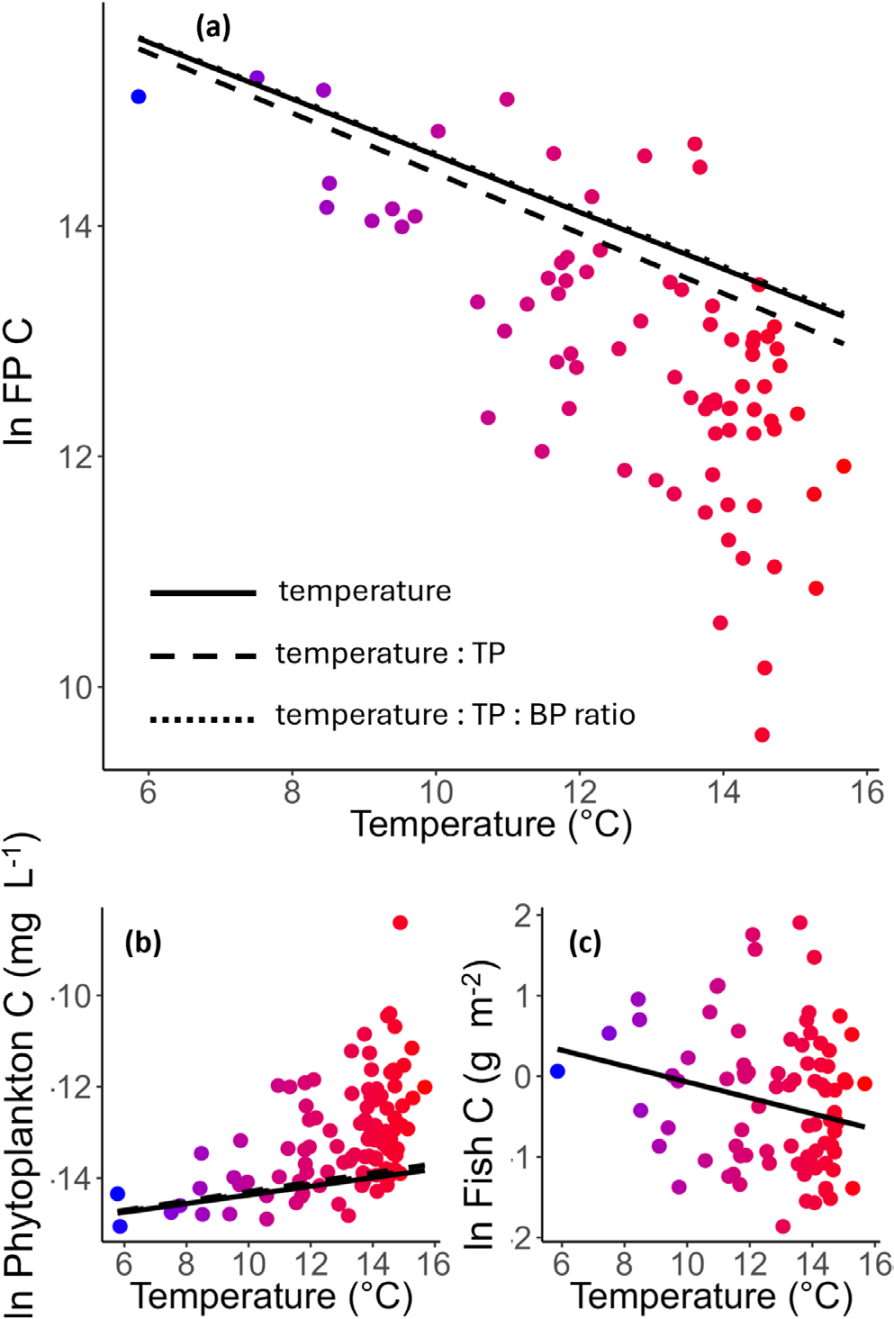
Relationships between temperature and (a) the ln ratio of fish‐phytoplankton (F/P) carbon biomass, (b) ln phytoplankton carbon biomass, and (c) ln fish carbon biomass per unit effort. Solid lines show the main effect of temperature; dashed lines indicate the temperature effect including the temperature × total phosphorus (TP) interaction; and dotted lines represent the temperature effect including the three‐way interaction between temperature, TP, and the benthic/pelagic (B/P) production ratio. Lines reflect significant model predictions (*p* < 0.05) from linear models (Table 1). Differences in slope among line types illustrate how the relationship between temperature and biomass shifts depending on nutrient availability and the B/P production ratio. Point colours represent mean air temperature, from blue (colder lakes) to red (warmer lakes).

The ratio of fish to phytoplankton biomass (F/P biomass ratio) is lower in warmer than in colder lakes (Figure 2a, Table 1). This is true irrespective of phosphorus concentration and B/P production ratio. However, the steepness of this decline increases at higher phosphorus concentrations (Figure S1), indicating that in more nutrient‐rich lakes, higher temperatures are associated with an even greater reduction in fish biomass relative to phytoplankton (see the steeper dashed line in Figure 2a). This effect of phosphorus is, however, counteracted by an increase in the B/P production ratio, which decreases the magnitude of the decline in the F/P biomass ratio with temperature caused by high phosphorus concentrations (see the shallower dotted line in Figure 2a). Temperature, and its interaction with phosphorus and the B/P production ratio, collectively explained 70% of the variance in the F/P biomass ratio (Table 1). Irrespective of model setup, most of the variation around the mean occurs among warmer lakes (Figure 2a), but with model residuals still adhering to assumptions of normality and heteroscedasticity (Figure S5a).

**TABLE 1:** Outputs of the linear models: *lm(*response variable ~ temperature + temperature: TP + temperature: TP: B/P production ratio).

Response variable	Explanatory variable	Estimate	SE	*t*‐value	*p*‐value	R^2^	Adjusted R^2^	*N*
ln F/P ratio	(Intercept)	17.066	0.485	35.214	3.13E‐49***	0.702	0.69	81
	Temperature	−0.246	0.04	−6.124	3.59E‐08***			
	Temperature: TP	−0.015	0.002	−8.231	3.65E‐12***			
	Temperature: TP: B/P production ratio	0.017	0.002	7.05	6.68E‐10***			
ln phytoplankton biomass	(Intercept)	−15.3	0.358	−42.8	2.26E‐67***	0.771	0.765	107
	Temperature	0.095	0.029	3.3	1.33E‐03***			
	Temperature: TP	0.008	0.001	8.67	6.74E‐14***			
	Temperature: TP: B/P production ratio	−0.004	0.001	−3.49	7.04E‐04***			
ln fish CPUE	(Intercept)	0.914	0.569	1.608	0.112	0.112	0.08	88
	Temperature	−0.098	0.046	−2.144	0.035*			
	Temperature: TP	−0.001	0.002	−0.727	0.469			
	Temperature: TP: B/P production ratio	0.003	0.002	1.254	0.213			

*Note:* The response variables include fish to phytoplankton biomass ratio (F/P ratio), and fish catch‐per‐unit‐effort biomass (Fish CPUE). All biomass values are expressed as carbon mass. Significance codes: < 0.001***, < 0.01**, < 0.05*.

The change in F/P biomass ratio with temperature is caused by temperature effects on both phytoplankton and fish. The biomass of phytoplankton is higher in warmer than in colder lakes, and more so in lakes with high phosphorus concentrations (Figure 2b, Table 1). There was also a significant interaction between temperature, phosphorus concentration, and the B/P production ratio on phytoplankton biomass. The positive effect of temperature on phytoplankton biomass is stronger at high phosphorus concentrations, whereas the inclusion of the B/P production ratio as a three‐way interaction decreases the magnitude of the response. Temperature, phosphorus, and the B/P production ratio collectively explained 77% of the variance in phytoplankton biomass (Table 1). In contrast to phytoplankton, fish CPUE was somewhat lower in warmer compared to colder lakes (Figure 2c, Table 1). This temperature effect on fish CPUE did not change with phosphorus concentration or B/P production ratio and explained only 8% of the variation in fish CPUE (Table 1). This discrepancy between the positive relationship between phytoplankton and temperature, combined with the negative response of fish CPUE, results in a decline in the F/P biomass ratio in warmer relative to colder lakes (Figure 2, Table 1).

### 3.1 Trend Lake Analysis

**FIGURE 3:**
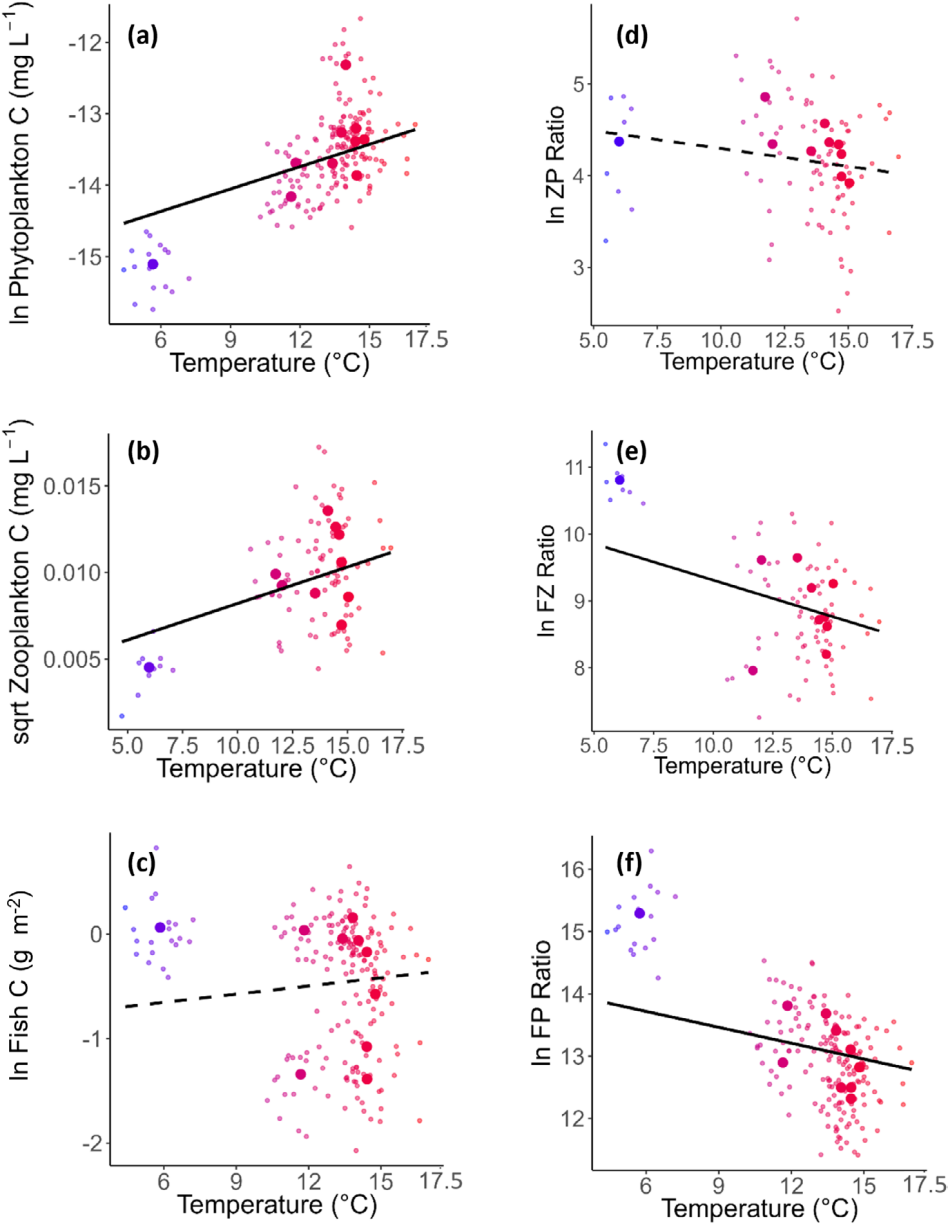
The relationship between temperature and (a) ln phytoplankton carbon biomass, (b) square root zooplankton carbon biomass, (c) ln fish carbon biomass per unit effort (CPUE), (d) ln zooplankton‐phytoplankton carbon biomass ratio, (e) ln fish‐zooplankton carbon biomass ratio and (f) ln fish‐phytoplankton carbon biomass ratio. Data are from the 10 trend lakes sampled from 2014 to 2023. Each point is plotted using the mean air temperature recorded over the productive period (May to September) during the same year the biomass data were collected. Smaller points represent annual means, and larger points represent mean values across the entire time period for each lake. Solid lines reflect significant relationships (*p* < 0.05), and dashed lines indicate non‐significant relationships from linear mixed effect models (Table 2). Point colours represent mean air temperature, from blue (colder lakes) to red (warmer lakes).

The temporal analysis, using annual biomass estimates across trophic levels in 10 trend lakes, revealed the same temperature effects on phytoplankton biomass and on the F/P biomass ratio as in the spatial analysis across 81 lakes (Figure 3, Table 2). Phytoplankton biomass showed a clear positive relationship with temperature (Figure 3a), but with a substantial proportion of the variance explained by the random effect of lake. In contrast, there was no effect of temperature on fish CPUE (Figure 3c, Table 2), and a larger proportion of the variance was explained by the random effect of lake. This indicates little to no temporal change in fish CPUE in response to changes in yearly growth season temperatures in the studied time period but change in phytoplankton biomass within lakes due to temperature variation. The trend lake F/P biomass ratios were negatively related to temperature (Figure 3f, Table 2), as in the main analysis.

**TABLE 2:** Outputs of the linear mixed effect models: Lme(response variable ~ temperature, random = ~ 1 + year | lake).

Response variable	Explanatory variable	Estimate	SE	*t*‐value	*p*‐value	*R* ^2^m	*R* ^2^c	*N*
ln phytoplankton biomass	(Intercept)	−14.993	0.399	−37.57	0.000***	0.167	0.768	186
	Temperature	0.104	0.028	3.679	0.000***			
Sqrt zooplankton biomass	(Intercept)	0.004	0.003	1.548	0.126	0.139	0.594	94
	Temperature	0	0	2.215	0.030*			
ln fish CPUE	(Intercept)	−0.81	0.374	−2.168	0.031*	0.01	0.851	200
	Temperature	0.026	0.024	1.071	0.286			
ln Z/P ratio	(Intercept)	4.685	0.43	10.89	0.000***	0.129	0.027	89
	Temperature	−0.039	0.032	−1.21	0.229			
ln F/Z ratio	(Intercept)	10.398	0.713	14.59	0.000***	0.117	0.675	82
	Temperature	−0.109	0.052	−2.09	0.040*			
F/P ratio	(Intercept)	14.229	0.57	24.97	0.000***	0.703	0.068	185
	Temperature	−0.085	0.041	−2.08	0.039*			

*Note:* The response variables include fish catch‐per‐unit‐effort biomass (Fish CPUE), zooplankton to phytoplankton biomass ratio (Z/P ratio), fish to zooplankton biomass ratio (F/Z ratio), and fish to phytoplankton biomass ratio (F/P ratio). All biomass values are expressed as carbon mass. Significance codes: < 0.001***, < 0.01**, < 0.05*.

To pinpoint where most energy losses occur in warmer lakes (i.e., indicated by the lower F/P biomass ratio), we also analysed zooplankton biomass, zooplankton to phytoplankton (Z/P) and fish to zooplankton (F/Z) biomass ratios using the trend lake data. There was a weak positive effect of temperature on zooplankton biomass (Figure 3c), but with most of the variance being explained by the random effect of lake (Table 2). There was no effect of temperature on the Z/P biomass ratio (Figure 3b), but a negative effect on the F/Z biomass ratio (Figure 3e). In other words, there is less fish CPUE relative to zooplankton biomass in lakes of higher temperatures (Figure 3, Table 2).

To test the robustness of these patterns, we conducted a sensitivity analysis where we removed the coldest and northernmost lake (Abiskojaure; Table S1). This removal did not alter the direction or significance of temperature effects on phytoplankton biomass or the F/P biomass ratio, but did alter the relationships involving zooplankton biomass and the F/Z ratio to non‐significant (Table S10).

## 4 Discussion

Our analysis of 125 temperate and sub‐arctic lakes across a wide temperature gradient reveals a shift in biomass distribution across trophic levels. In contrast to our hypothesis, fish biomass was lower relative to phytoplankton biomass in warmer compared to colder lakes. Specifically, phytoplankton biomass is significantly higher in warmer relative to colder lakes. This relationship holds when accounting for between‐lake variation in nutrient availability and the relative proportion of benthic to whole lake primary productivity. This finding contrasts several earlier experimental studies (Bideault et al. 2021; O'Connor et al. 2009; Shurin et al. 2012, but see Nagelkerken et al. 2020) and a few observational studies (e.g., Ishikawa et al. 2023 on plankton communities) that suggest more top‐heavy biomass pyramids in warmer food webs. A higher biomass of pelagic primary producers should intuitively support a higher biomass of fish through improved food availability. The lack of such an effect in our study suggests reduced energy transfer from pelagic primary producers to higher trophic levels at warmer temperatures.

Increased phytoplankton biomass in warmer lakes may, based on previous findings, be explained by a positive relationship between temperature, individual metabolic rates, and growth rates of phytoplankton (Bernhardt et al. 2018). However, it remains uncertain how biomass is ultimately affected when increased phytoplankton productivity driven by higher temperatures is offset by reduced carrying capacity and increased consumption rates (Boyd et al. 2013; Yvon‐Durocher et al. 2011). Still, if accelerated rates of photosynthesis and reproduction at higher temperatures (Brown et al. 2004; López‐Urrutia et al. 2006) lead to greater phytoplankton production, this may translate to a greater standing stock biomass. Warmer conditions may, however, not only change overall primary production and biomass, but also tend to favor smaller, faster‐growing phytoplankton species (Sommer and Lengfellner 2008; Zohary et al. 2021). These smaller and warm‐adapted species are often of lower food quality (Gobler 2020; Jöhnk et al. 2008; Lau et al. 2021; O'Neil et al., 2012; Paerl and Huisman 2009), sometimes with a higher toxin content (Wilson et al. 2006) and a lack of essential lipids (Martin‐Creuzburg and Von Elert 2009). Such traits can reduce grazing efficiency by zooplankton (Burkholder et al. 2018), leading to a build‐up of uneaten phytoplankton (Sommer and Lengfellner 2008). In combination with the rapid proliferation of these taxa in warm waters, this may explain the overall higher phytoplankton biomass observed in warm compared to cold lakes. This pattern was further supported by our supplementary Detrended Correspondence Analysis (DCA, Figure S2a), which revealed a shift in phytoplankton community composition along the temperature gradient (Figure S2b, Table S7). A regression between temperature and DCA axis 1 indicates that warmer lakes are associated with phytoplankton communities increasingly dominated by less edible taxa such as cyanobacteria. These taxa had high scores on DCA axis 1, which appears to reflect a gradient in phytoplankton edibility. Correspondingly, phytoplankton edibility was low in the warmest lakes (Figure 3a), which might contribute to explaining the observed decrease in the fish‐to‐phytoplankton biomass ratio across the temperature gradient from cooler to warmer lakes. This may suggest that a shift in phytoplankton taxa constrains the transfer of energy from phytoplankton to zooplankton and fish. However, phytoplankton edibility was also low in some of the cooler lakes (Figure S3a), suggesting that additional factors may be important, such as a relationship between temperature and consumer community composition.

Among zooplankton, we found that community biomass slightly increased with temperature. This contrasts with studies suggesting declines in zooplankton due to higher fish predation, in turn, caused by a positive relationship between temperature and feeding rates (unless very high temperatures; Beaugrand et al. 2002; He et al. 2020; Uszko et al. 2017), or due to phenological mismatches with phytoplankton (Edwards and Richardson 2004; He et al. 2020; Richardson 2008). These contrasting results indicate context‐dependent responses, which may include variation in food chain length (Hansson et al. 2013) and statistical limitation due to a low number (10) of lakes sampled for zooplankton. Of these 10 trend lakes, there was no relationship between temperature and zooplankton community composition, including small‐bodied lipid‐poor rotifers vs. larger‐bodied and more energy‐rich cladocerans and copepods (Figure S2c). This suggests that variation in zooplankton composition and therefore their nutritional value is unlikely to contribute much towards the negative relationship between temperature and the ratio of fish to phytoplankton biomass. Other factors such as direct physiological responses in fish may therefore be more important in limiting fish biomass despite high phytoplankton biomass in warm lakes.

The observed decline in fish biomass with increasing temperature, despite a substantial increase in phytoplankton biomass, may be attributed to three mechanisms. First, it may indicate indirect temperature effects mediated by shifts in prey community composition, rather than a decrease in overall prey availability. This interpretation is supported by the higher zooplankton biomass observed in the warmer lakes in our study (Figure 3b), although it contrasts with the absence of temperature‐related changes in zooplankton community composition in the trend lakes (Figure S2d). The lower fish biomass may also be linked to temperature‐dependent physiological responses, such as increased metabolic costs for fish in warmer environments, which means that more energy is required to sustain high body growth and survival (Atkinson 1994; Pörtner and Farrell 2008). This is particularly true for larger individuals (Lindmark, Ohlberget, et al. 2022), and warming can therefore induce size shifts towards smaller individuals (Arranz et al. 2023; Lindmark, Audzijonyte, et al. 2022; van Dorst et al. 2019). Given that species interactions are highly size‐dependent in aquatic food webs, such size shifts induced by warming can alter both predator–prey size ratios and the strength of trophic couplings (Gårdmark and Huss 2020), which may weaken the efficiency of energy transfer through food webs. While we observed a slight increase in zooplankton biomass in warm compared to cold lakes, it could be speculated that bottom‐up processes, specifically a reduction in the quality of zooplankton as food for fish, may result in the metabolic demands of fish not being met. However, the analyses of the limited number of lakes for which zooplankton data were available showed no change in zooplankton community composition. Instead, the clear shift in fish community composition with temperature (Figure S2f, Table S7) and a corresponding decline in mean trophic level in warmer lakes (Figure S3b, Table S8) suggest that in warmer lakes there are: (1) fewer trophic linkages, possibly reflecting a collapse of higher trophic levels due to reduced energy transfer efficiency, and (2) a dominance of small planktivorous fish. While these species can exploit zooplankton, they may not sustain the same biomass or support higher trophic level production to the same extent as cold‐water fish populations. Together these shifts can help explain the lower fish biomass in warmer lakes, despite increased primary producer biomass.

It is important to contextualise these findings concerning temperature dependencies. The temperature gradient in this study, while significant in range, does not encompass very warm lakes, in which phytoplankton production, for instance, may be less limited by temperature than in our study region (Bergström et al. 2013). In regions with higher growing‐season temperatures, such as tropical or subtropical areas, phytoplankton production might instead be more limited by nutrient availability (Fernández‐González et al. 2022; López‐Sandoval et al. 2021). Testing the generality of the temperature dependence of lake phytoplankton biomass, and therefore fish to phytoplankton biomass ratios, across additional climatic zones is therefore a critical next step. In the context of energy flows, trophic linkages, and resulting biomass distributions, it is also important to acknowledge that we cannot exclude the possibility that the observed shifts across study lakes may partially relate to organisms' groups and factors for which we had no data, had unrepresentative data, and/or did not include in our analyses for other reasons. For instance, we lacked data on macrophytes, which may influence fish habitat use and behaviour, and thereby affect local fish biomass estimates. Such habitat‐mediated behavioural responses could contribute to variability in observed biomass patterns. Also, there is an evident overrepresentation of southern relative to northern Swedish lakes, leading to greater representation of relatively warm and nutrient‐rich lakes.

We also observed greater between‐lake variation in nutrient concentrations and biomasses of different organism groups among the warmer lakes. Despite not violating the assumption of homoscedasticity, this variability suggests that additional factors not accounted for here may play a more significant role in the dynamics of warmer and nutrient‐rich lakes relative to colder and nutrient‐poor lakes. Furthermore, our focus was primarily on pelagic elements of primary producers and their interactions with zooplankton and fish, but we recognize benthic energy mobilization also plays a crucial role in lakes, especially in northern clear‐water lakes (Ask et al. 2009; Karlsson et al. 2015). We partly took this into account by including estimates of benthic to whole lake primary production ratios, which may influence overall biomass ratios and energy transfer. Additionally, we did not have access to data on bacteria and protozoans in our study lakes, which can be important energy sources for some meso‐zooplankton species and constitute additional trophic levels (Berglund et al. 2007; Cotner and Biddanda 2002; Work and Havens 2003). Finally, to infer the role of a potential relationship between temperature and energy transfer efficiency for biomass distributions across thermal gradients, we would need production estimates (Mehner et al. 2022), which were unavailable for this study. In light of these potential data limitations and the key role of northern lake ecosystems in ecosystem service provisioning, such as to carbon burial and release (Heathcote et al. 2015), we call for lake monitoring programs to include more cold, oligotrophic lakes, as well as sampling of benthic energy mobilizers.

Our results, based on a space‐for‐time approach including 125 lakes, suggest there is potential for a significant shift in biomass distributions in lakes from fish to phytoplankton as temperate, boreal, and sub‐arctic lakes become warmer. We propose changes in phytoplankton and fish community composition as potential mechanisms explaining why increased phytoplankton biomass in warmer waters does not propagate up the food web to fuel higher fish biomass. Declines in higher trophic level biomass, including economically important fish species, can affect water quality (Bernes et al. 2013) and local fishing opportunities (Christensen et al. 2003). Shifts from fish towards phytoplankton‐dominated food webs may also modify top‐down control and therefore disrupt food web dynamics and stability. The fact that phytoplankton can apparently benefit from warmer waters much more than consumers, combined with earlier findings of increased input and cycling of nutrients at high temperatures (Meerhoff et al. 2022), suggests that warming may exacerbate symptoms of eutrophication in lakes. In nutrient‐rich waters, this could cause shading of benthic habitats, hypoxia, and negative impacts on aquatic biodiversity (Anderson et al. 2008; Diaz and Rosenberg 2008), although the extent to which warming‐induced eutrophication would lead to those same consequences is less known. Our findings on shifts in biomass distributions call for further investigations to pinpoint the direction and cause of change in biomass ratios over thermal gradients in a range of study systems, as well as the extent to which our space‐for‐time predictions hold also in the case of actual warming. In conclusion, our study highlights a critical shift in biomass distribution from fish to phytoplankton along thermal gradients, which may indicate future declines in aquatic food web efficiency and predator biomass under climate warming.

## Author Contributions

**Benjamin Paul Mooney:** conceptualization, data curation, formal analysis, investigation, methodology, visualization, writing – original draft. **Anna Gårdmark:** conceptualization, methodology, supervision, writing – review and editing. **Carolyn Faithfull:** conceptualization, methodology, writing – review and editing. **Renee Mina van Dorst:** methodology, writing – review and editing. **Magnus Huss:** conceptualization, funding acquisition, methodology, supervision, writing – review and editing.

## Conflicts of Interest

The authors declare no conflicts of interest.

## Supporting information


Data S1.

## Data Availability

The data that support the findings of this study are available in Dryad at https://doi.org/10.5061/dryad.51c59zwkx. Fish data were obtained from the Swedish University of Agricultural Sciences, Department of Aquatic Resources at https://aquarapport.slu.se/. Phytoplankton, zooplankton and water geochemical data were obtained from the Miljödata MVM database at https://miljodata.slu.se/MVM/Search. Air temperature was obtained from the Swedish Meteorological and Hydrological Institute at https://www.smhi.se/data/ladda‐ner‐data/griddade‐nederbord‐och‐temperaturdata‐pthbv/.
