# Supplementary material for: Warmer Lakes Support Phytoplankton Over Fish
Source: Glob Chang Biol. 2025 Jun 9;31(6):e70288. doi: 10.1111/gcb.70288 (PMC12147057; doi:10.1111/gcb.70288)
Supplement: Supplementary file 1 — Data S1. [file GCB-31-e70288-s001.docx]

Supplementary information for the study:

Warmer Lakes Support Phytoplankton Over Fish

Benjamin Paul Mooney^1^, Anna Gårdmark^1^, Carolyn Faithfull^1^, Renee Mina van Dorst^2^, Magnus Huss^1^

1. Swedish University of Agricultural Sciences, Department of Aquatic Resources, SLU, SE-750 07, Uppsala, Sweden
2. Swedish University of Agricultural Sciences, Department of Wildlife, Fish and Environmental Studies, SLU, SE- 901 83, Umeå, Sweden

Supplementary methods and analyses

Additional Data on Zooplankton Biomass

To address the gap in data for lakes with intermediate temperatures in the trend lake analysis, where zooplankton biomass was compared across lakes of varying temperatures over a 12-year period, we incorporated additional zooplankton biomass data reported in the supplement of Bergström et al. (2022). We chose to include data from this paper because it includes pelagic zooplankton data from Swedish boreal lakes of low to intermediate temperatures, which are underrepresented in our main dataset. Carbon mass was converted from the reported dry mass by a factor of 0.45 (Jaspers et al., 2009). Air temperatures for these lakes were calculated using the same methodology as described in the main manuscript (see Materials and Methods, ‘Water Temperature’).

A linear model was then applied to evaluate whether the observed linear relationship between zooplankton biomass and lake temperature in the main study was driven by the single cold lake, or whether it remained consistent when data from lakes with intermediate temperatures were included. The direction and significance remained with the inclusion of these lakes (Figure S4a).

Community Composition

To better understand the mechanisms driving variation in the fish to phytoplankton (F/P) biomass ratio with temperature, we tested for the effects of phytoplankton, zooplankton and fish community composition. The rationale for investigating community composition is that some species of phytoplankton and zooplankton are more nutritious and/or edible than others (Lepori & Capelli, 2020), and that fish species occupy different trophic levels and may therefore utilise pelagic invertebrate resources more or less efficiently (i.e. planktivorous fish being more efficient). A higher mean trophic level of the fish community also implies additional steps of energy transfer from basal resources. Variation in community composition might therefore explain differences in either phytoplankton, zooplankton or fish biomass, or a combination of all three.

To test differences in community composition between phytoplankton, zooplankton and fish, we used Detrended Correspondence Analyses (DCA; Oksanen et al., 2022). For phytoplankton and fish, each observation was a lake, as per the main analysis. For zooplankton, each observation was an individual year from one of 10 lakes, as per the trend lake analysis. The first DCA axes of these analyses were then tested as response variables against the same explanatory variables as in the main analysis (this was the case for phytoplankton DCA1 and fish DCA1) and as in the trend analysis (this was the case for zooplankton DCA1). The response variables were inverse hyperbolic sine transformed due to negative values in the DCA axis values. These analyses were performed to identify whether there was a change in community composition with temperature. For the communities from all lakes included in the main analysis, phytoplankton DCA axis 1 explained 68.96% of the variation in the ordination and fish DCA axis 1 explained 78.85%. As for trend lake data, zooplankton DCA axes 1 explained 31.14% of the variation (Fig. S2).

Phytoplankton DCA1 (and therefore community composition) changed with temperature, with minor additional effects from interactions between temperature, nutrient availability and the relative contribution of benthic to pelagic primary production. Zooplankton DCA1 was not related to temperature, whereas fish DCA1 was related to temperature only (Fig. S2; Table S7).

Edibility and Trophic Position

To identify how lakes differ in overall edibility of the phytoplankton community, we assigned an edibility factor to each phytoplankton family (Table S5). We calculated the amount of edible biovolume per lake by multiplying the biovolume of each phytoplankton family by their edibility factor (Lepori & Capelli, 2020), and summing across all families. These values were subsequently divided by the total biovolume of phytoplankton in each lake to give a value of ‘edibility’ per lake.

To identify how lakes differ in mean trophic level of the fish community, species-specific trophic positions were extracted from the R-package rfishbase (N = 46, Boettiger et al., 2012). FishBase ‘FoodTroph’ trophic positions are estimates from a number of food items from different studies using a randomised resampling routine. These values were divided by the total biomass per unit effort of all fish in each lake to calculate the mean trophic value of fish per lake.
To test whether changes in phytoplankton and fish community composition are correlated with changes in edibility and mean trophic value, respectively, we conducted a polynomial regression between DCA1 axis of phytoplankton community composition and edibility, and another between the DCA1 axis of fish community composition and mean trophic value. The polynomial relationships in both models were significant. 24.40% of the variance in edibility is explained by phytoplankton DCA1, and 77.30% of the variance in mean trophic value is explained by fish DCA1 (Table S8).

Sensitivity Analysis

To evaluate the sensitivity of our findings given the potential leverage of a single lake in the temporal analysis of the 10 trend lakes, we tested the effect of removing the northernmost lake, which was also the coldest in our dataset (Abiskojaure, SE758208-161749; Table S1). The general direction of temperature effects remained consistent across trophic levels and the significance concerning the relative changes in phytoplankton and fish across the temperature gradient was retained. However, the relationships between temperature, zooplankton biomass and fish-to-zooplankton biomass became non-significant (Table S10). While these changes suggest some sensitivity of the analysis to the inclusion of extreme thermal conditions, they also indicate that our overall conclusions are not driven by a single outlying lake. Rather, they reflect broader temperature-dependent patterns across the study region.

Supplementary Figures


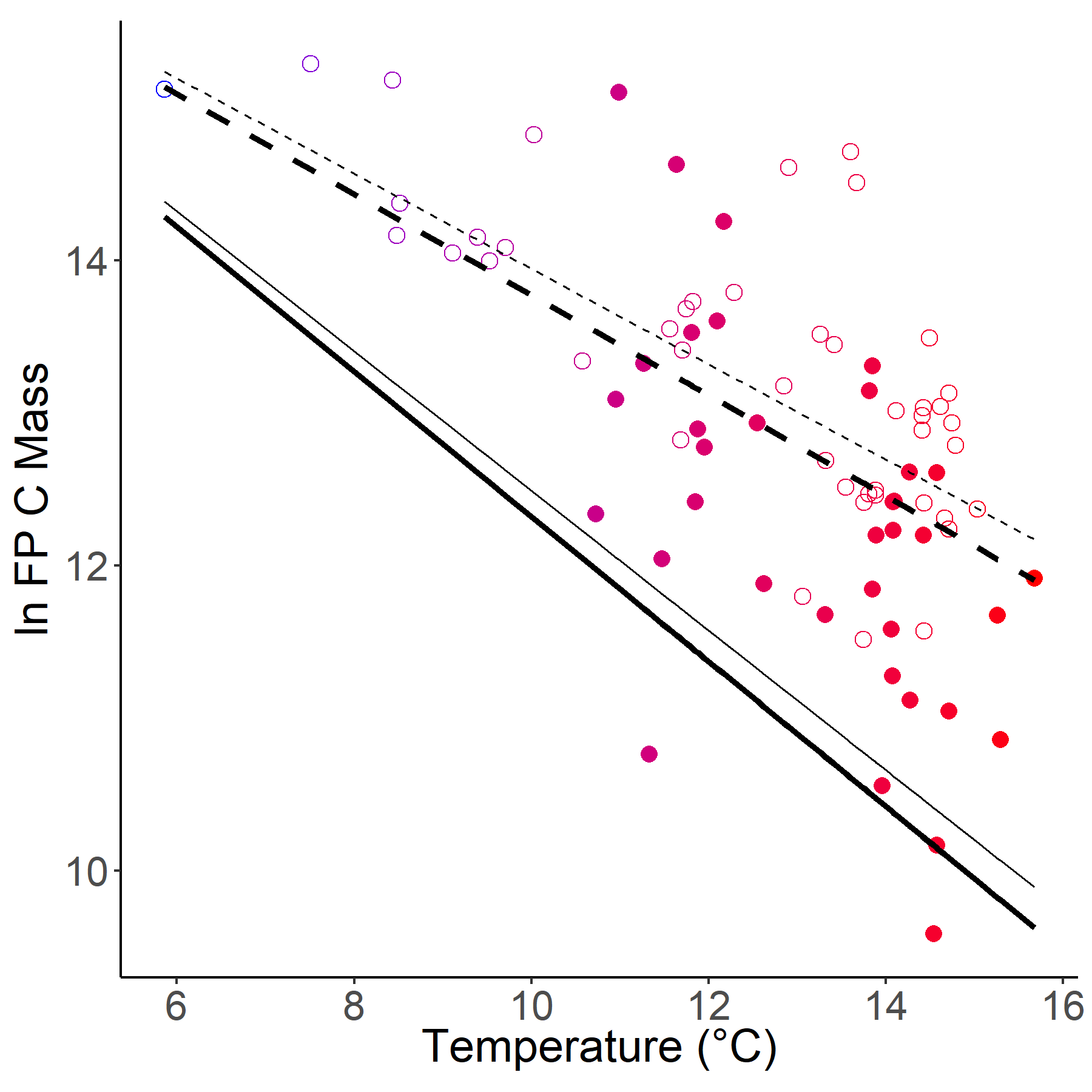


oligotrophic

meso/ eutrophic

temperature

temperature : BP ratio

**Figure S1.** Relationships between temperature and the natural log ratio of fish to phytoplankton (F/P) carbon. Bold lines represent model predictions based on temperature alone. Thinner lines indicate the interaction between temperature and benthic-to-pelagic production (BP) ratio. For plotting purposes only, lakes were categorised into oligotrophic (low total phosphorus; solid lines) and mesotrophic/eutrophic (high total phosphorus; dashed lines) based on a threshold of 10 µg L⁻¹ (Vollenweider & Kerekes, 1982). Lines are plotted based the median total phosphorus (TP) concentrations for each category, 5.49 µg L⁻¹ and 15.02 µg L⁻¹ respectively. Lines on the plot represent significant (P < 0.05) model predictions (see Table 1). Point colours represent mean air temperature, ranging from blue (colder lakes) to red (warmer lakes).


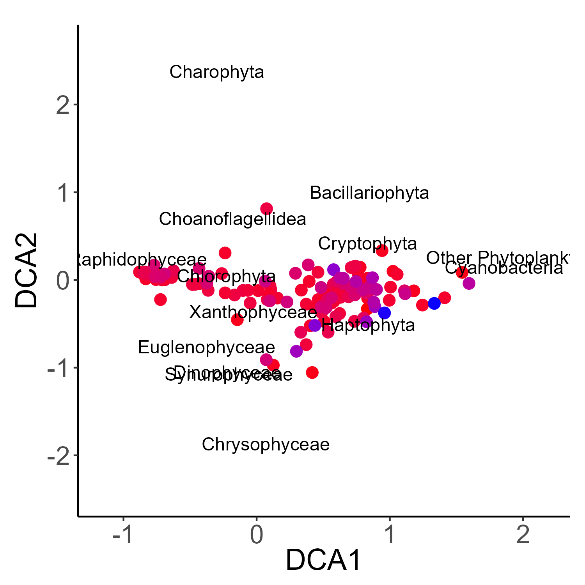

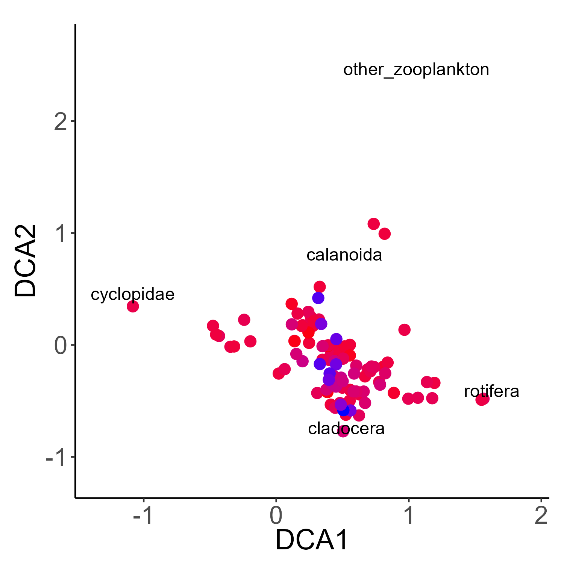

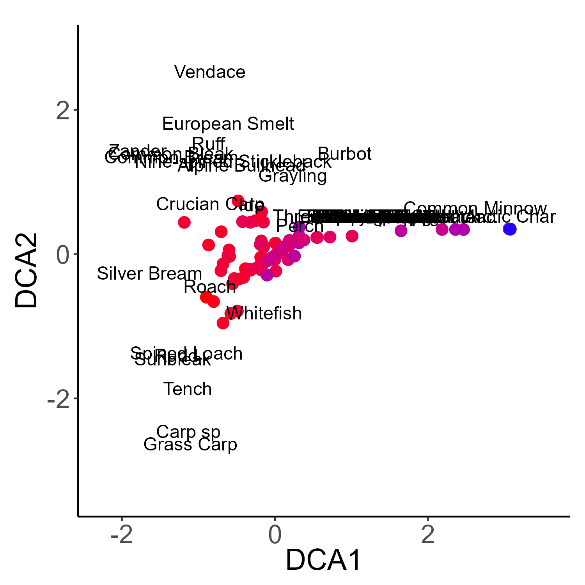

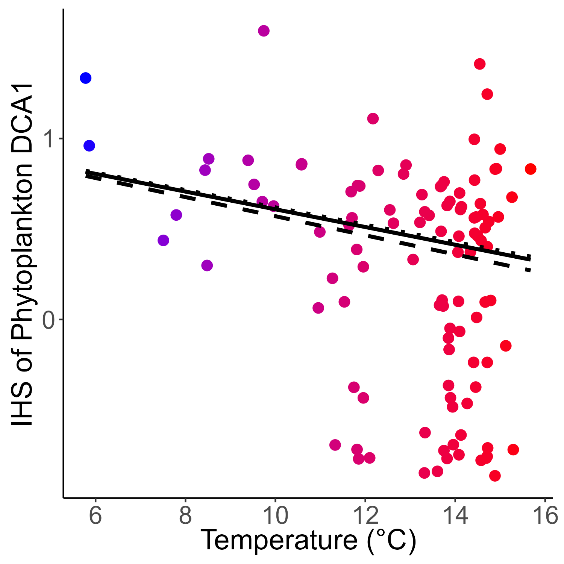

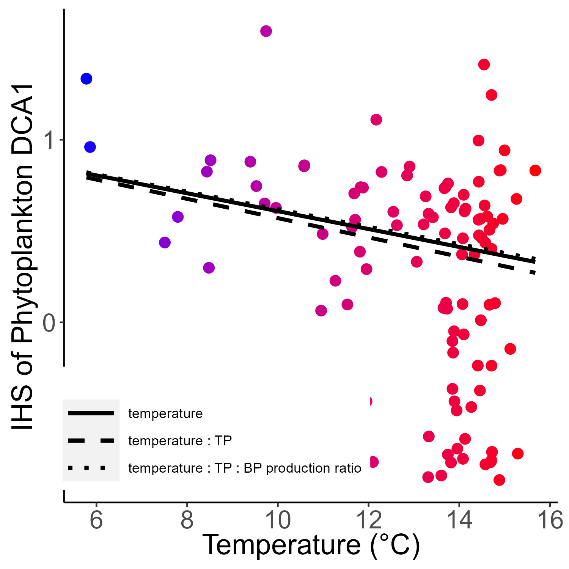

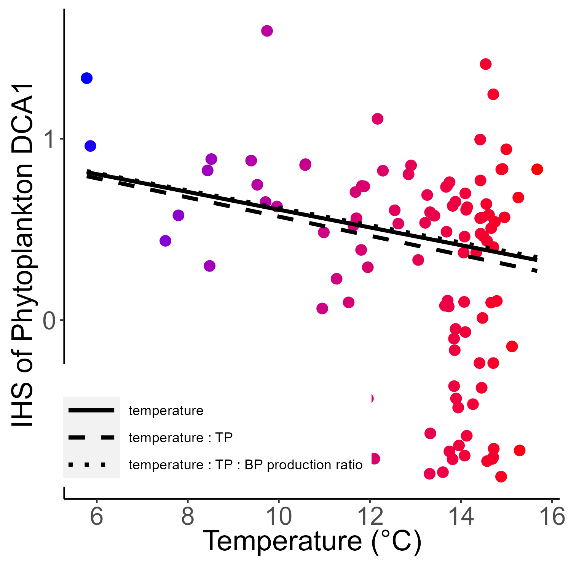

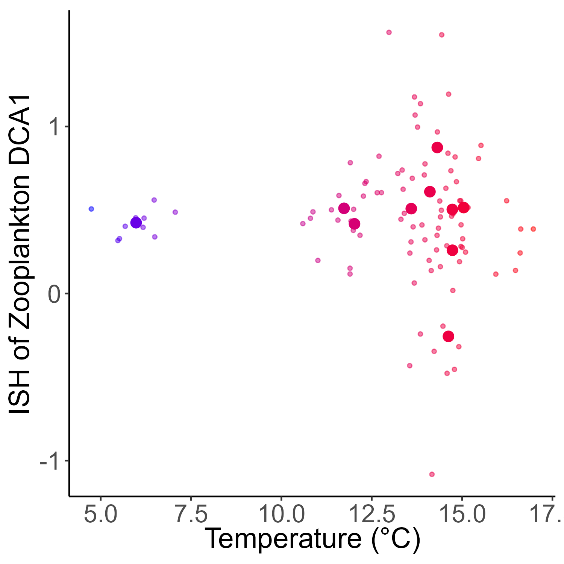


5


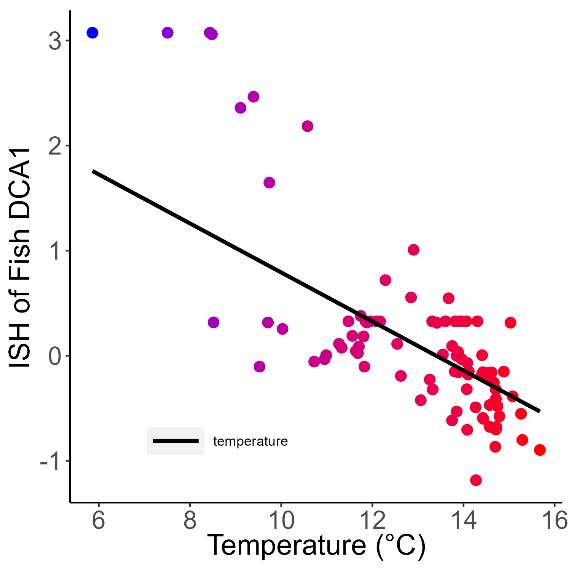

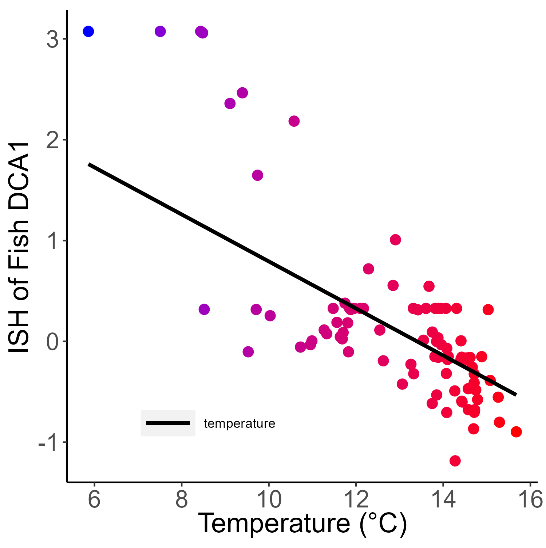


**(a)**

**(b)**

**(c)**

**(d)**

**(e)**

**(f)**

**Figure S2.** On the left, detrended Correspondence Analysis (DCA) of (a) phytoplankton community composition, (c) zooplankton community composition and (e) fish community composition. On the right, relationships between temperature and (b) the inverse hyperbolic sine of phytoplankton DCA axis 1, (d) the inverse hyperbolic sine of zooplankton DCA axis 1, and (f) the inverse hyperbolic sine of fish DCA axis 1. Solid lines show the main effect of temperature; dashed lines indicate the temperature effect including the temperature × total phosphorus (TP) interaction; and dotted lines represent the temperature effect including the three-way interaction between temperature, TP, and the benthic/pelagic (B/P) production ratio. Lines on the plot represent significant (P < 0.05) model predictions (Table S7). Point colours represent mean air temperature, ranging from blue (colder lakes) to red (warmer lakes).
**
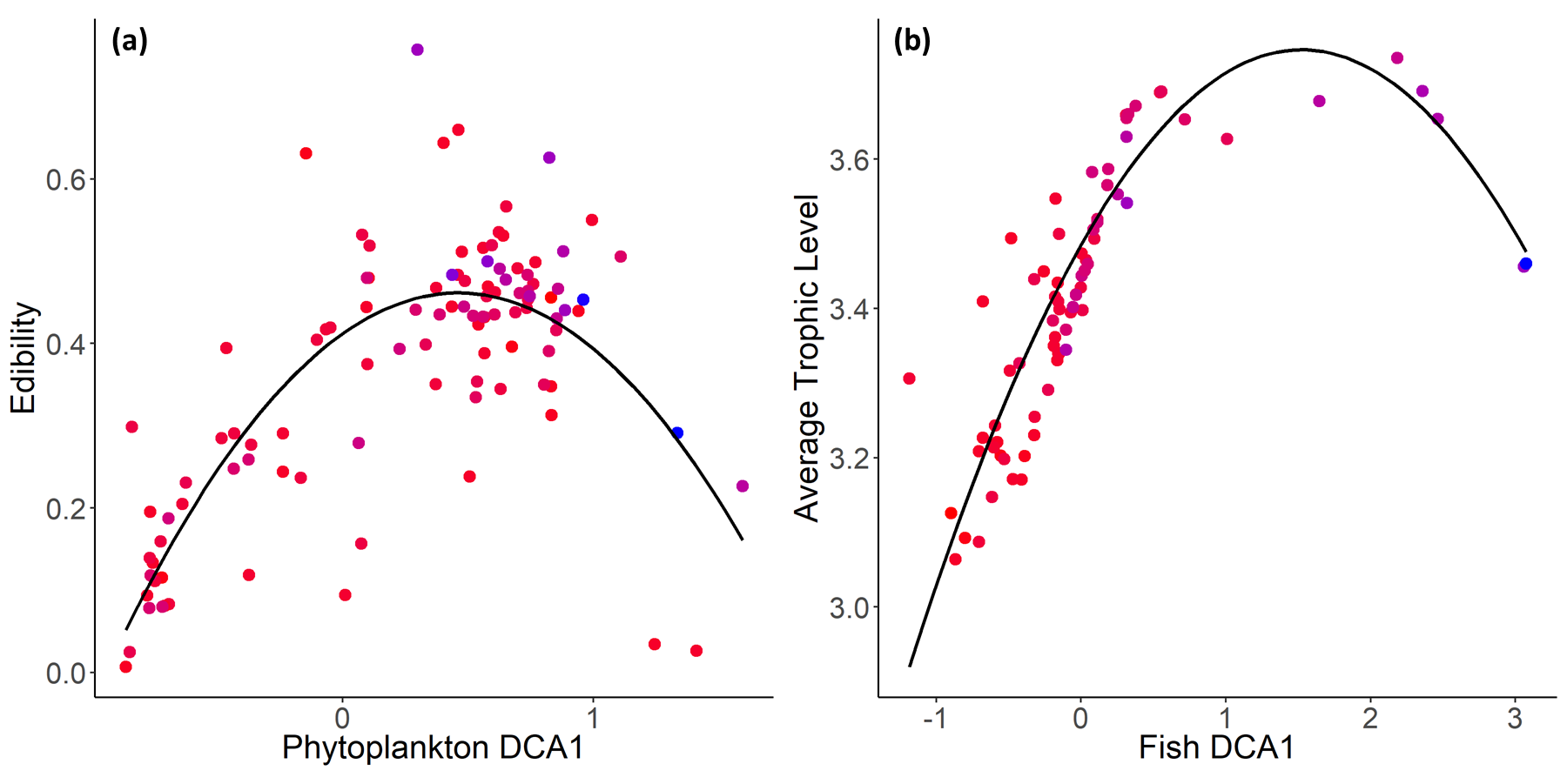
**

**Figure S3.** Polynomial regressions between (a) phytoplankton DCA axis 1 and phytoplankton community edibility and (b) fish DCA axis 1 and fish community mean trophic position. The community compositions that these DCA axes represent are shown in Fig S2a for phytoplankton and Fig S2e for fish. Lines reflect significant model predictions (P > 0.05) from linear models (Table S8). Point colours represent mean air temperature, from blue (colder lakes) to red (warmer lakes).

*
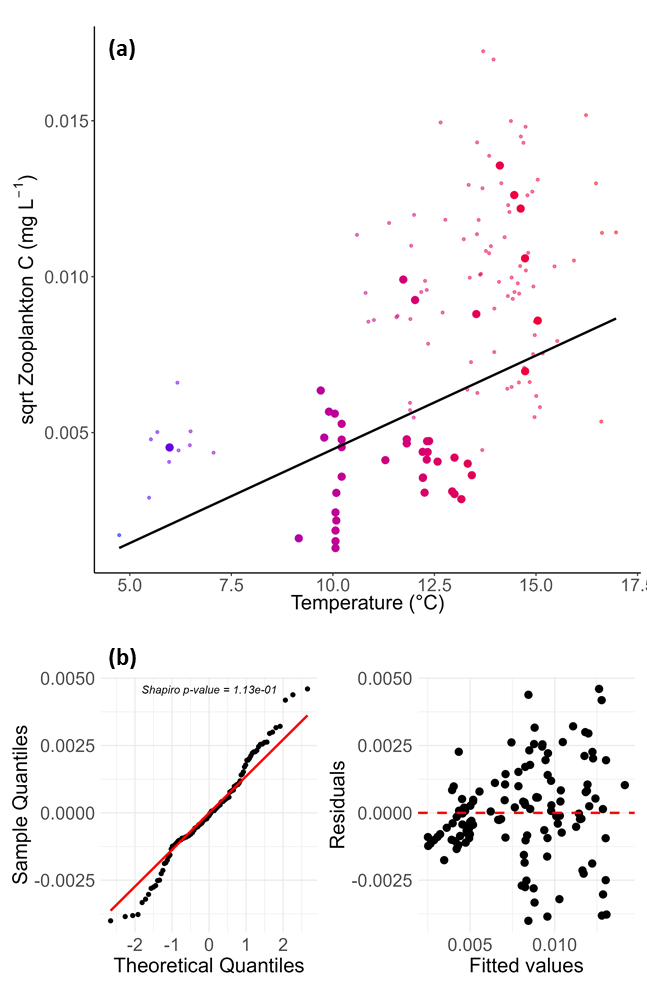
*

**Figure S4. (a)** The relationship between the sqrt zooplankton biomass and temperature. Data is for the 10 trend lakes from 2014 – 2023, and the mean air temperature recorded over the productive period (May to September) during each of those years. The smaller points are yearly means and large points are mean values over the entire time period for each lake. The large points circled in blue are data from Bergström et al., 2022. The solid line represents a significant relationship (p < 0.05). Point colours represent mean air temperature, from blue (colder lakes) to red (warmer lakes). **(b)** Residual analysis from this linear model. On the left are Q-Q plots of the residuals from the linear models. The red line represents the reference line for a normal distribution. On the right the residuals from the linear models are plotted against the fitted values. The red dashed line at zero indicates no residual deviation.


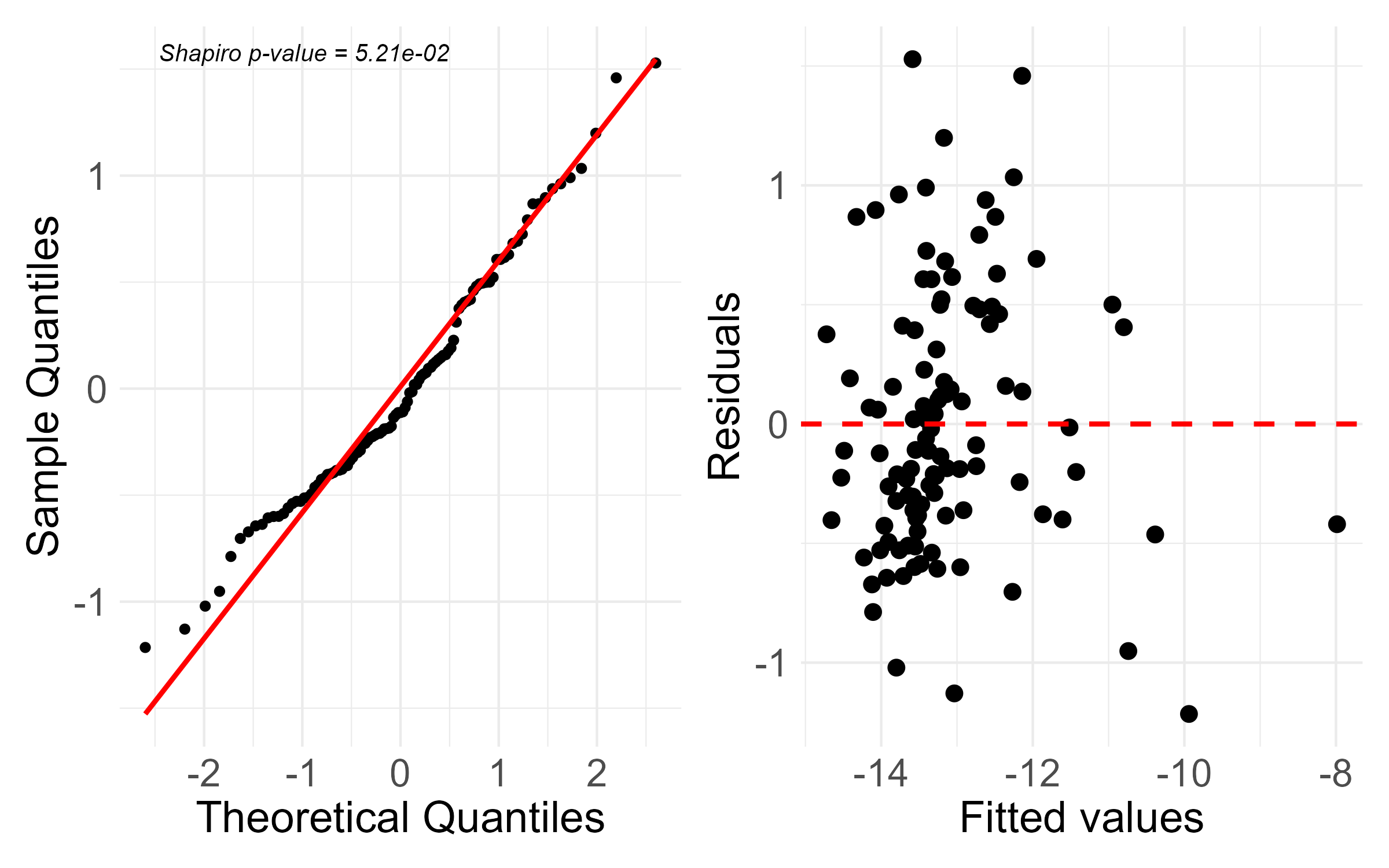

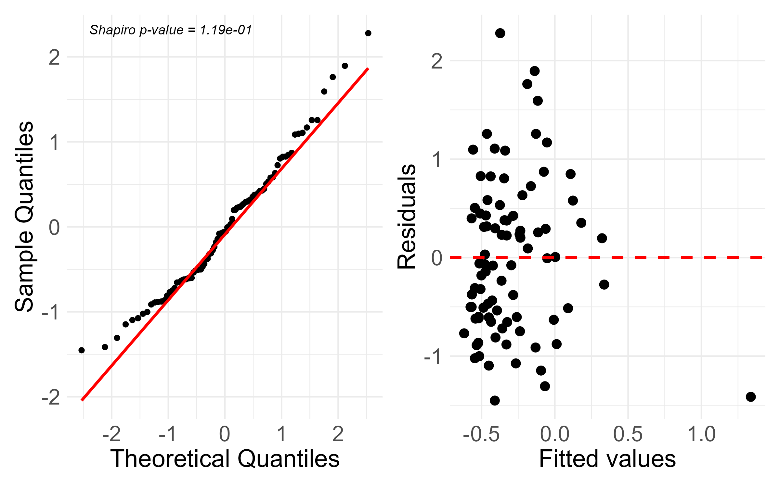

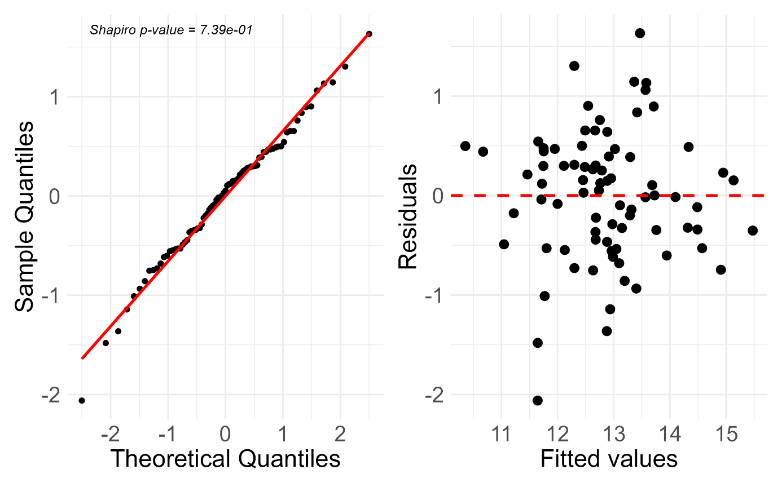


**(a)**

**(b)**

**(c)**


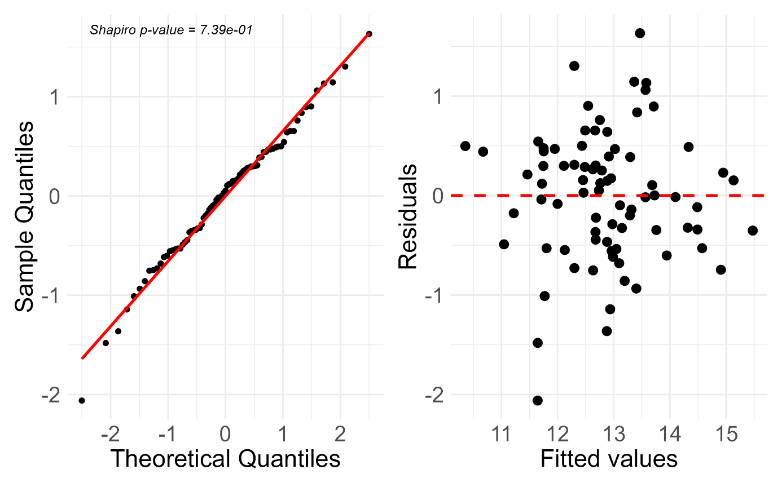

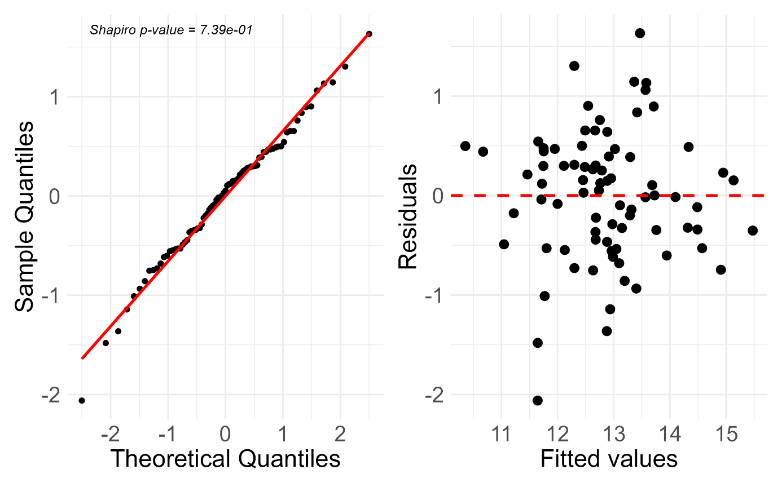

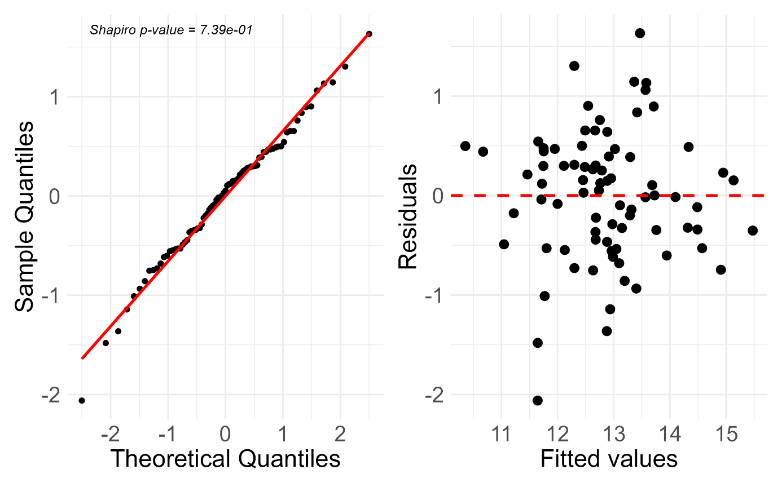


**Figure S5.** Residual analysis for the linear models: *lm(response variable ~ temperature + temperature : TP + temperature : TP : B/P production ratio* with the response variables (a) log_e_ fish to phytoplankton carbon biomass ratio, (b) log_e_ phytoplankton carbon biomass and (c) log_e_ fish carbon biomass. On the left are Q-Q plots of the residuals from the linear models. The red line represents the reference line for a normal distribution. On the right the residuals from the linear models are plotted against the fitted values. The red dashed line at zero indicates no residual deviation. TP stands for total phosphorus and B/P production ratio for benthic/ whole lake primary production ratio.


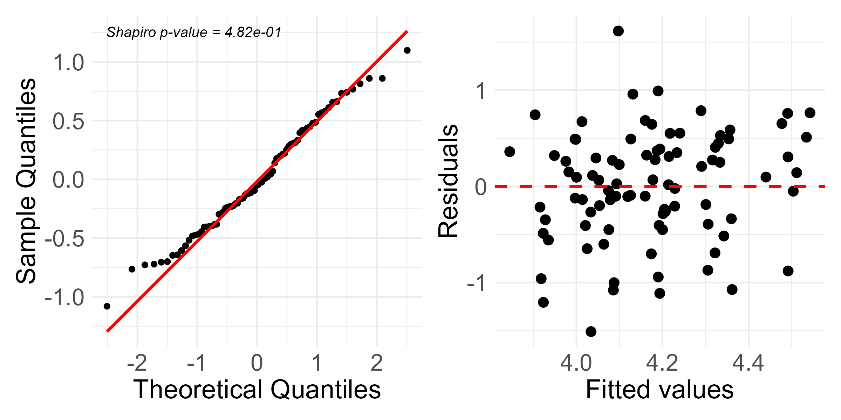

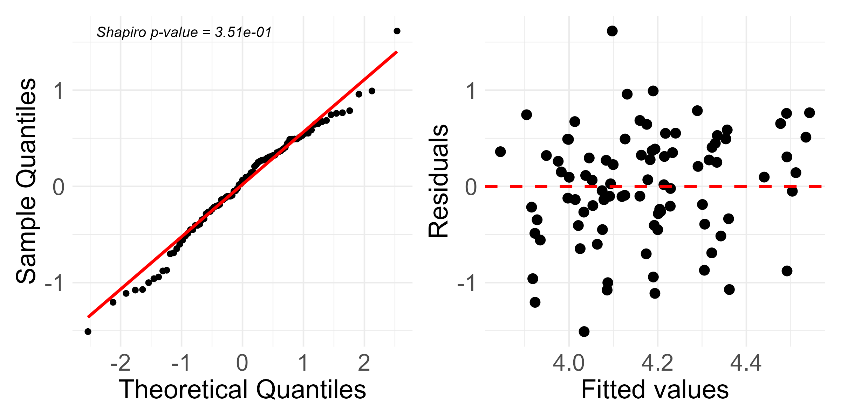

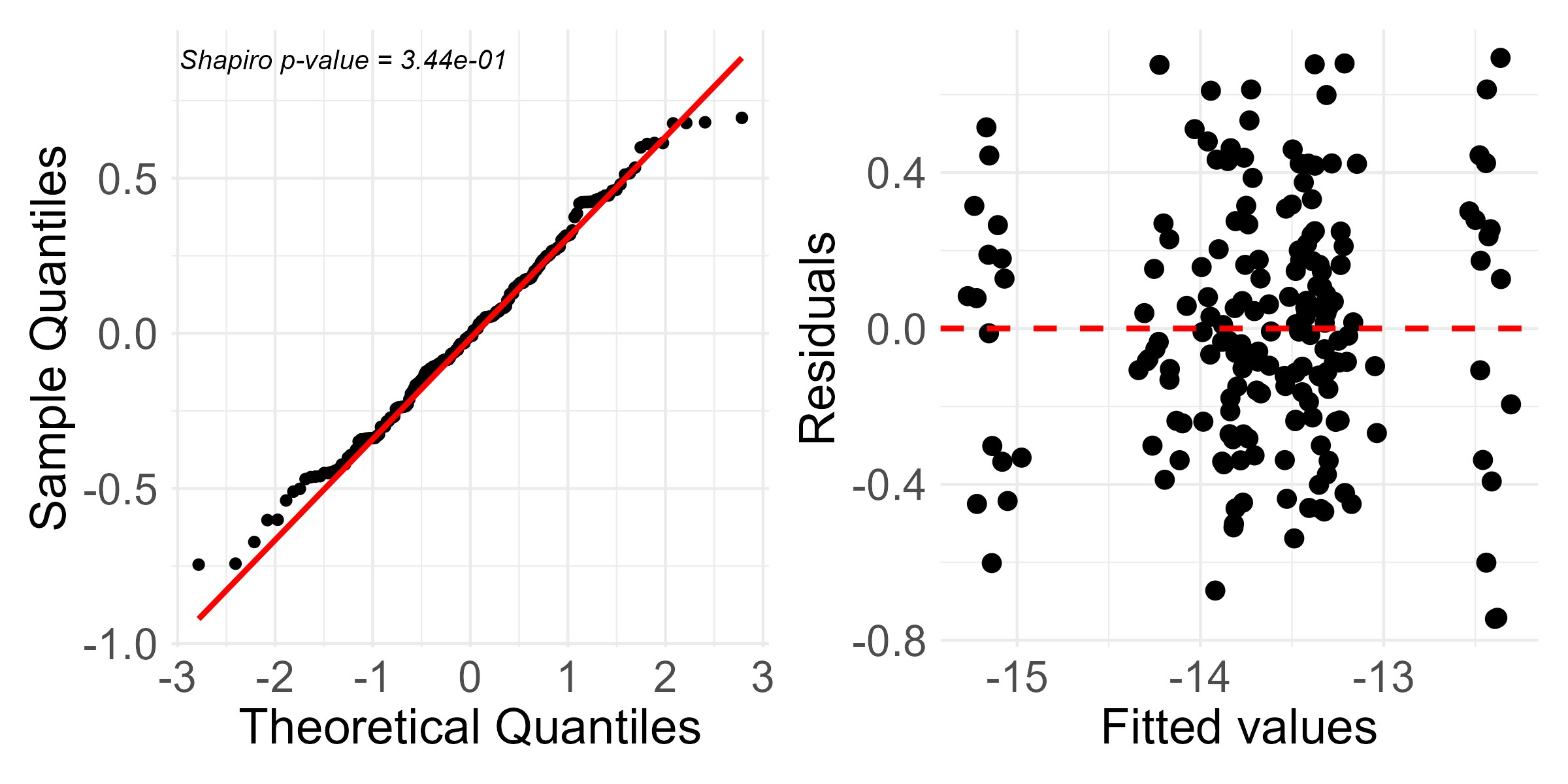

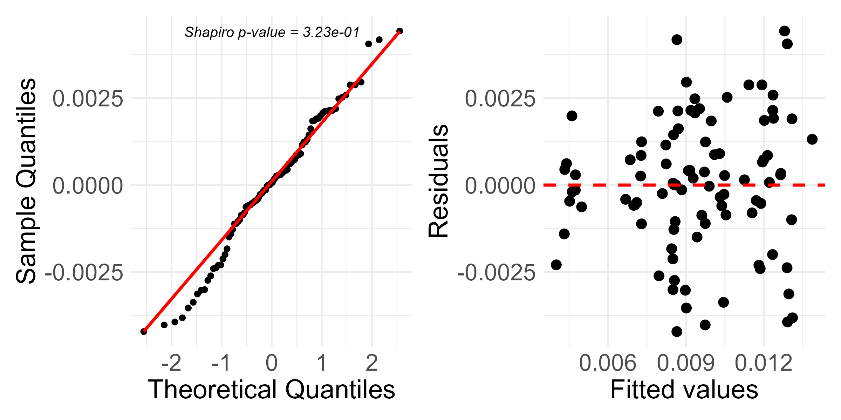

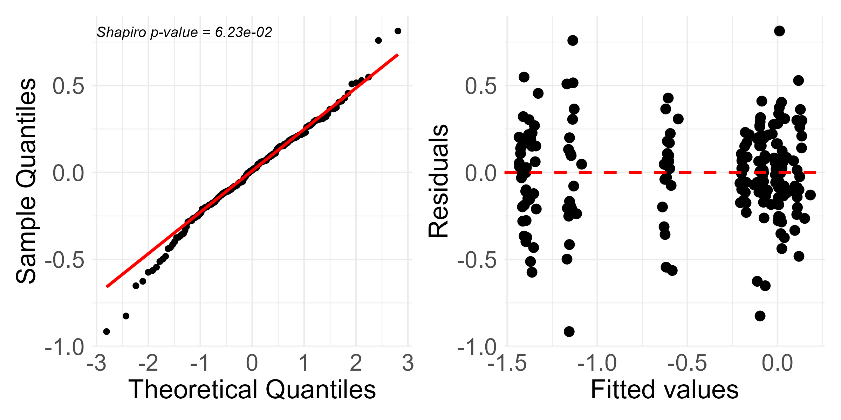


**(a)**

**(d)**

**(b)**

**(e)**

**(c)**


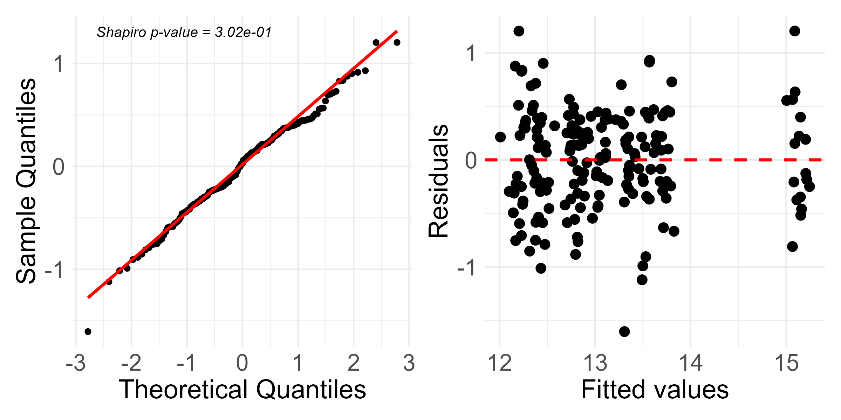


**(f)**


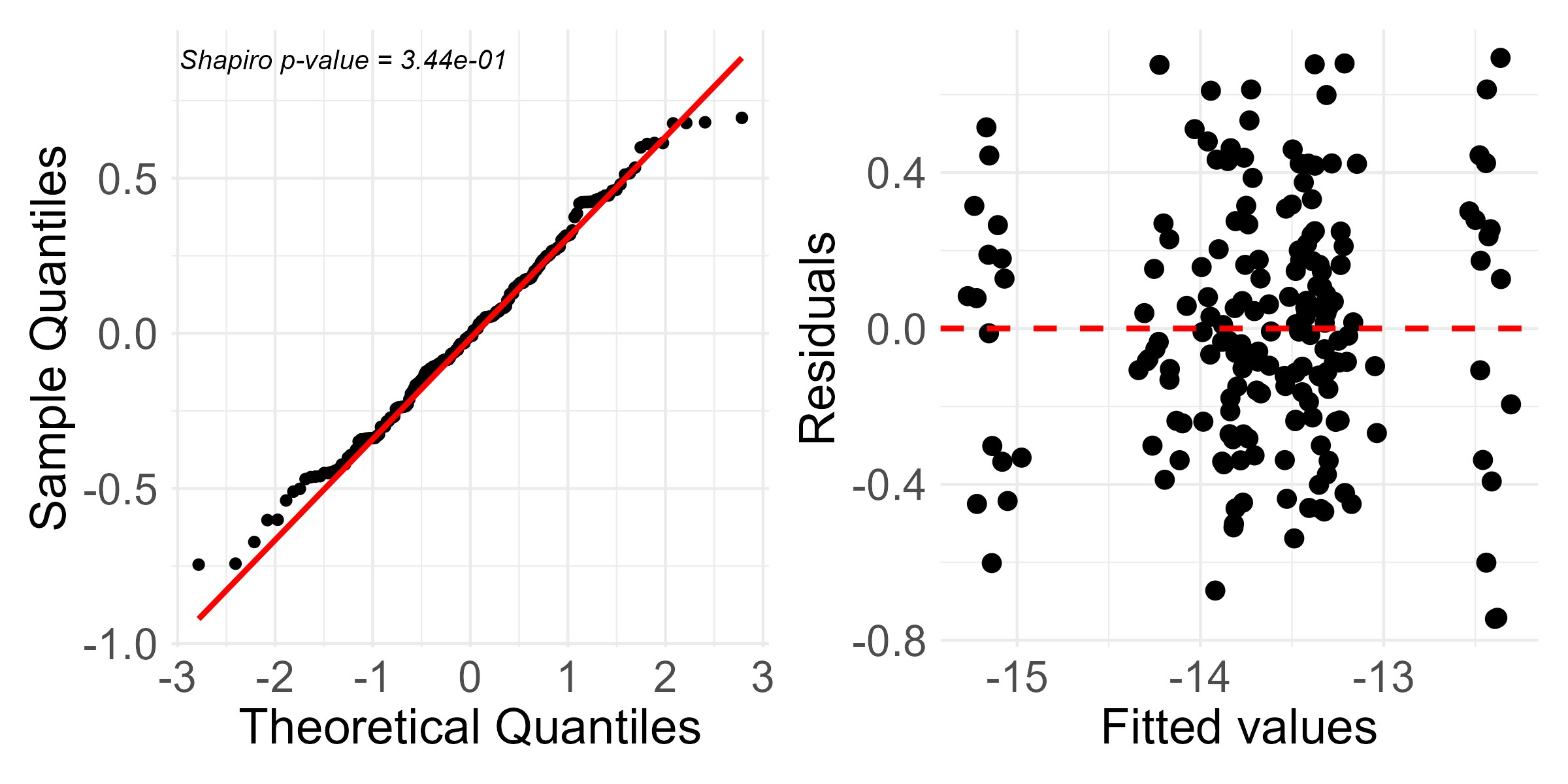

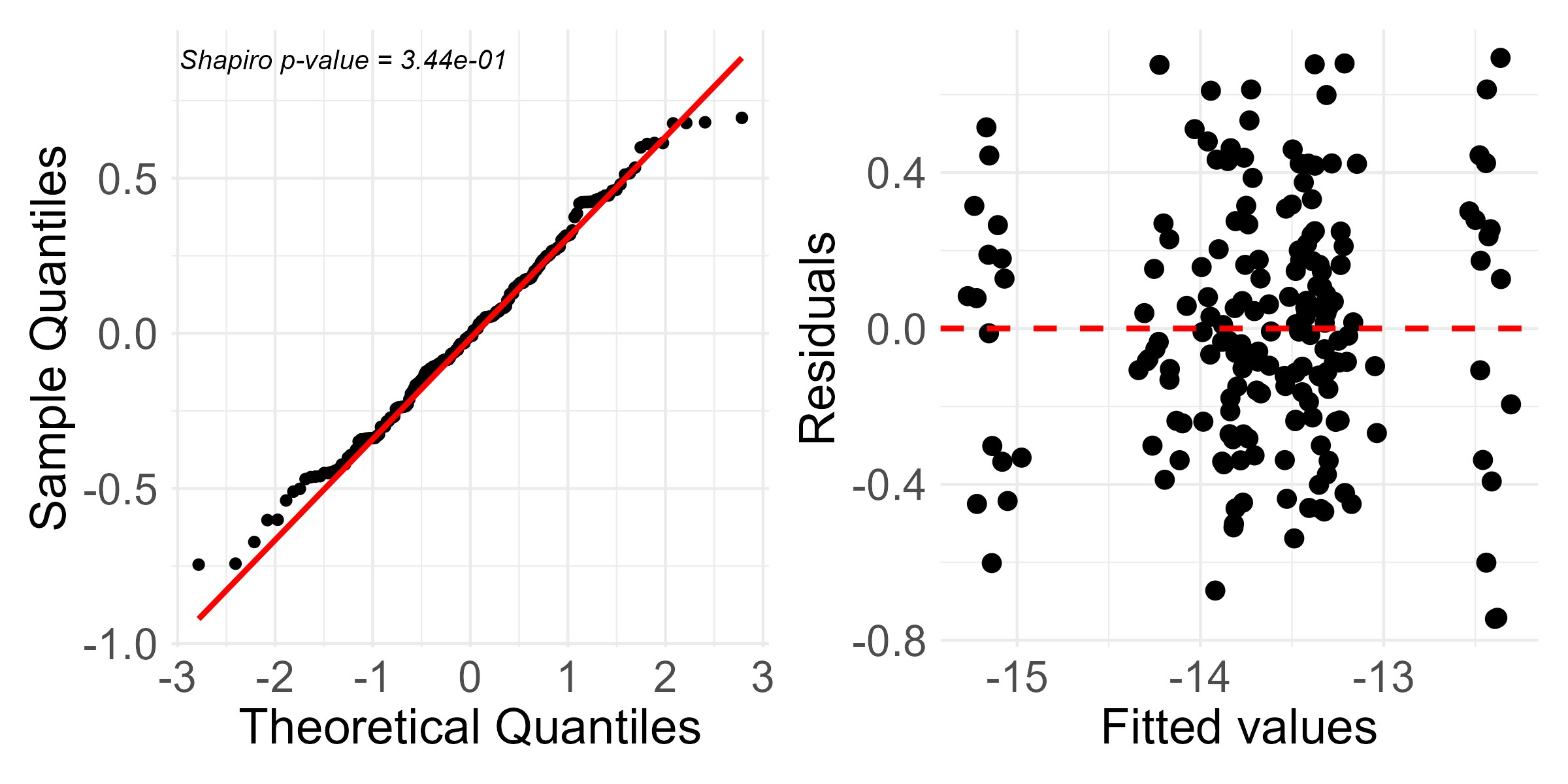

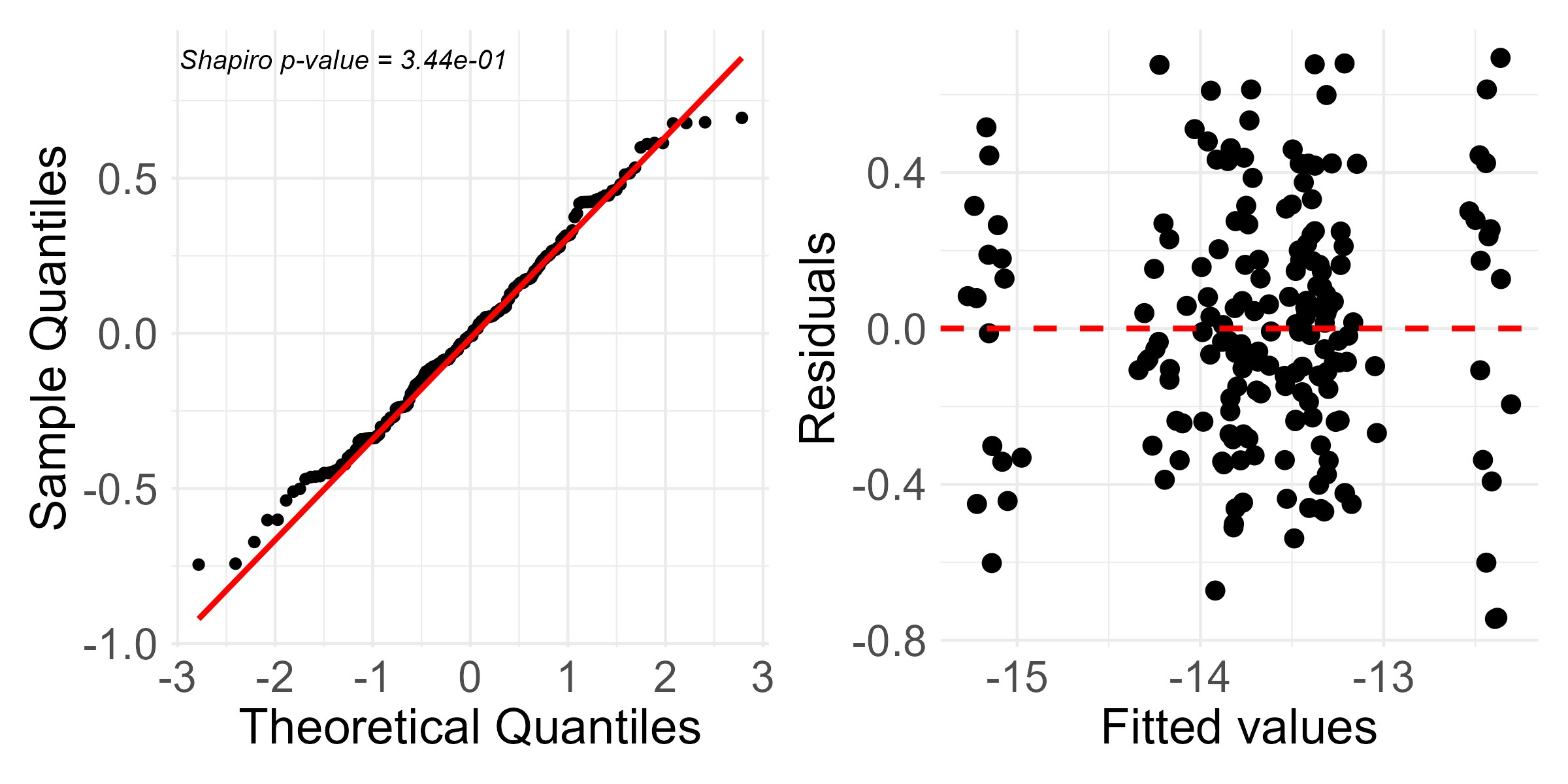

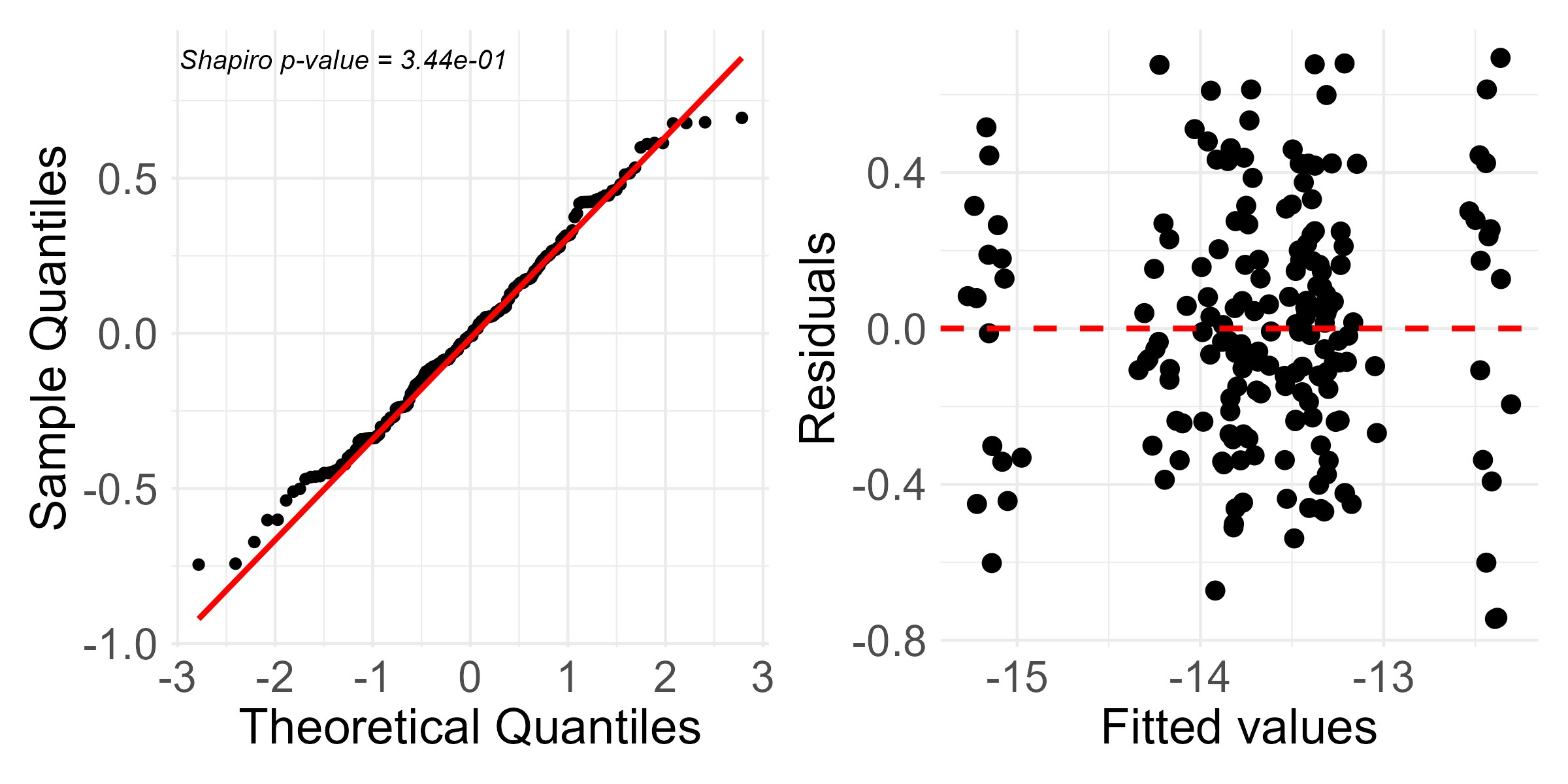

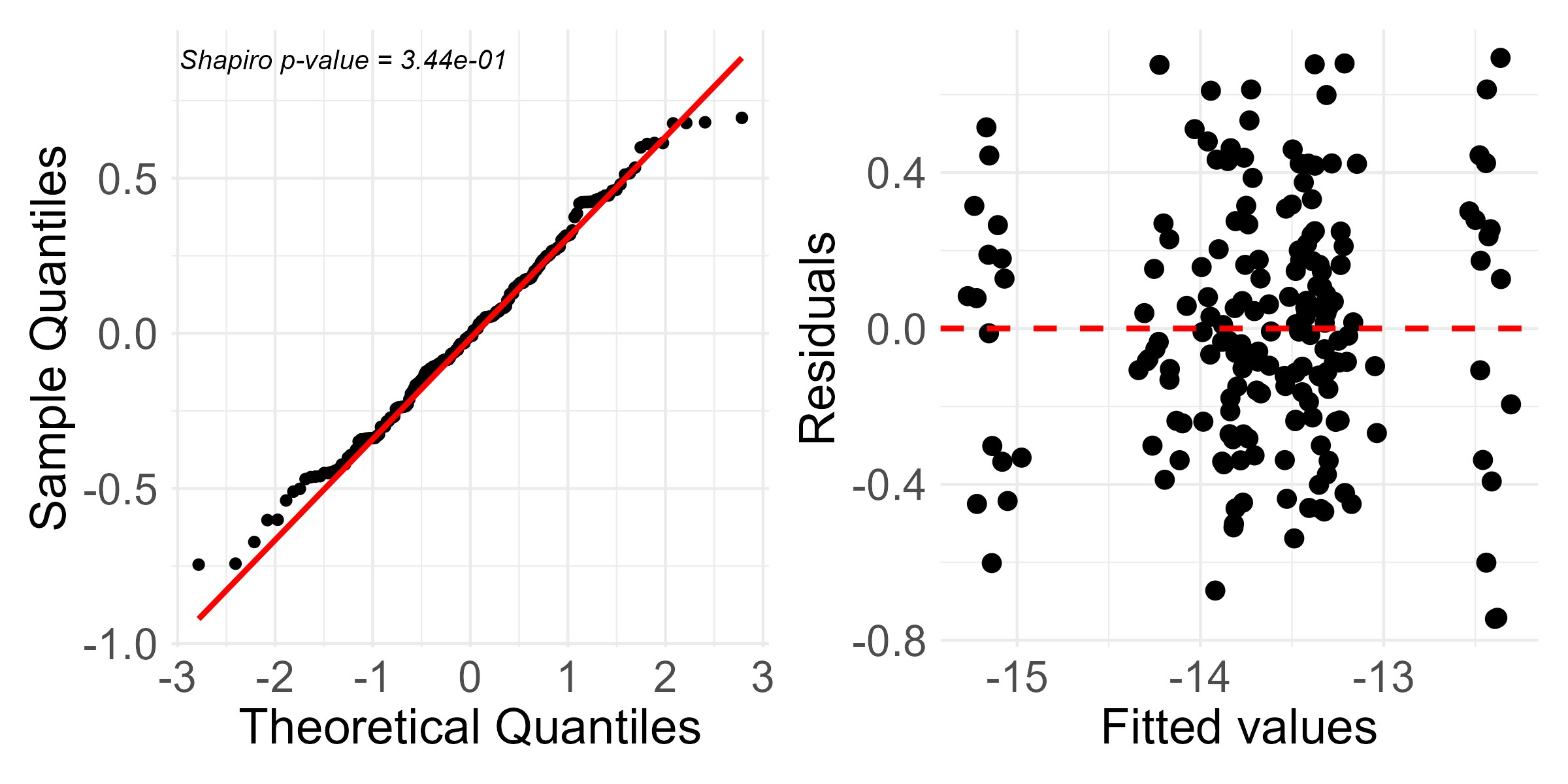

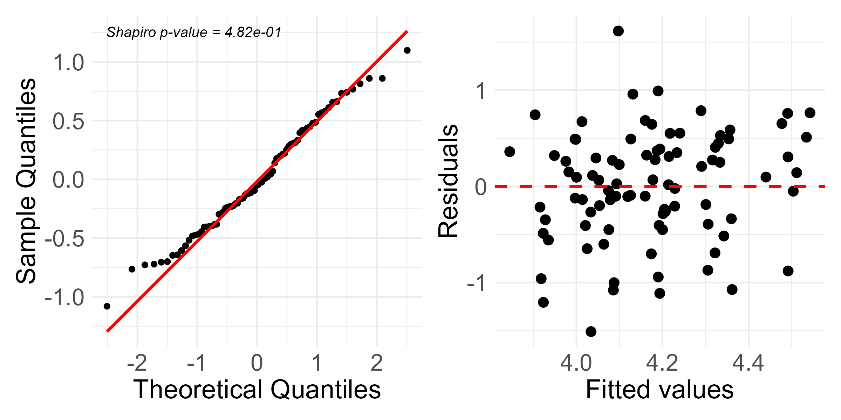


**Figure S6.** Residual analysis for the linear mixed effect models: *lme(response variable ~ temperature, random = ~ 1 + year | lake)* with the response variables (a) ln phytoplankton carbon biomass, (b) square root zooplankton carbon biomass, (c) ln fish carbon biomass per unit effort (CPUE), (d) ln zooplankton to fish carbon biomass ratio, (e) ln fish to zooplankton carbon biomass ratio and (f) ln fish to phytoplankton carbon biomass ratio. On the left are Q-Q plots of the residuals from the linear models. The red line represents the reference line for a normal distribution. On the right the residuals from the linear models are plotted against the fitted values. The red dashed line at zero indicates no residual deviation. TP stands for total phosphorus and B/P production ratio for benthic/ whole lake primary production ratio.


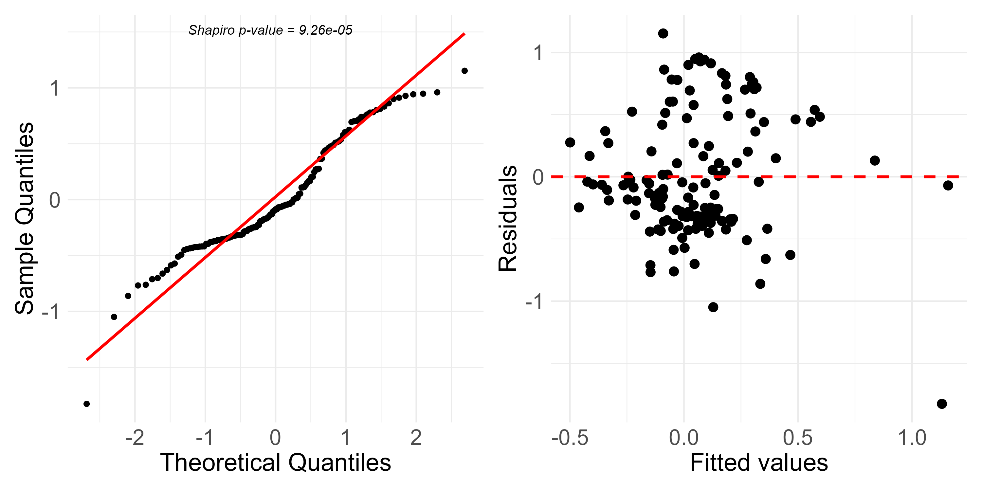

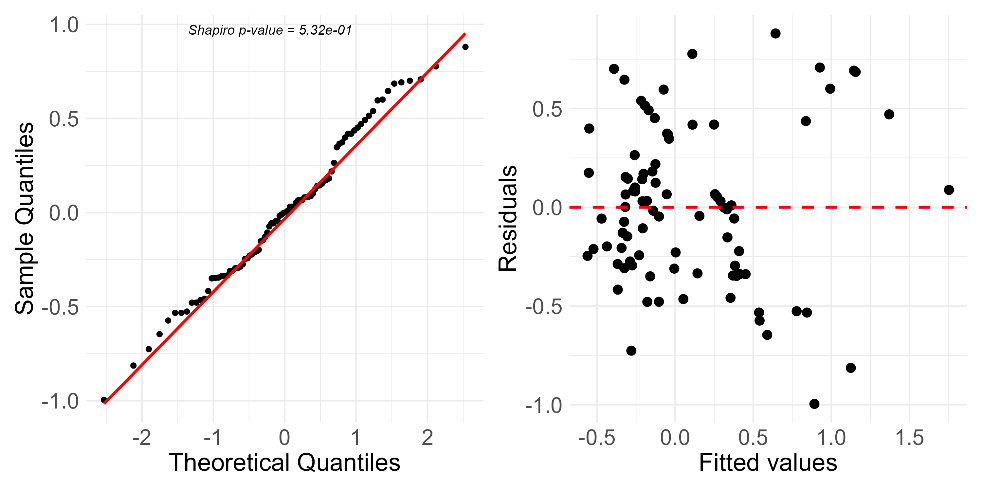

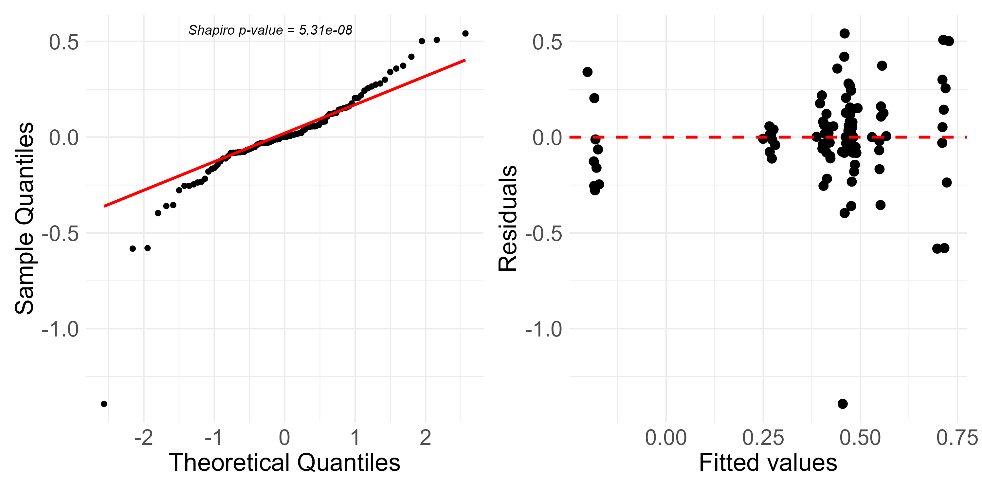


**(a)**

**(b)**

**(c)**


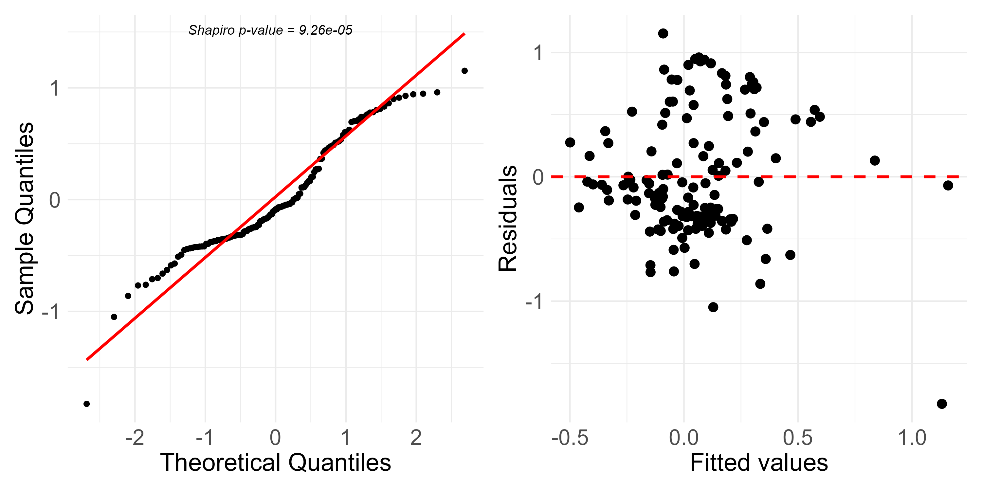

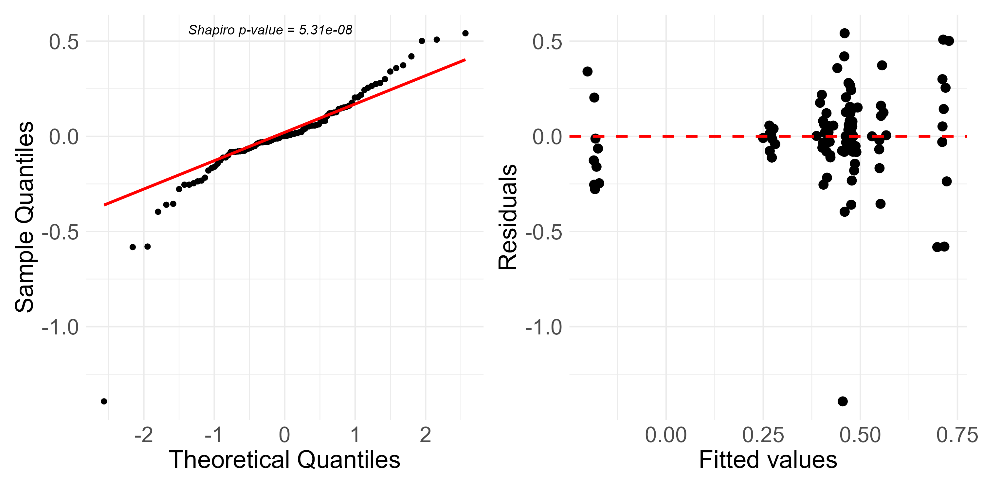

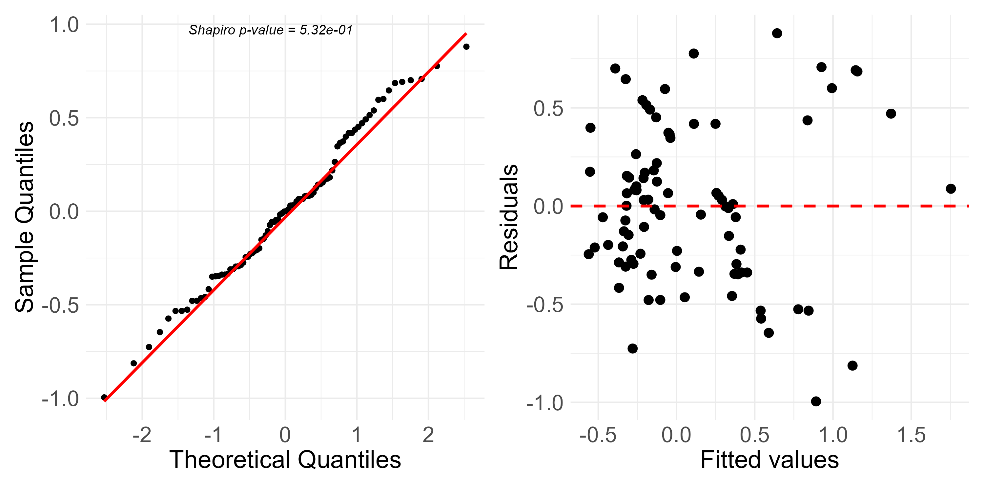

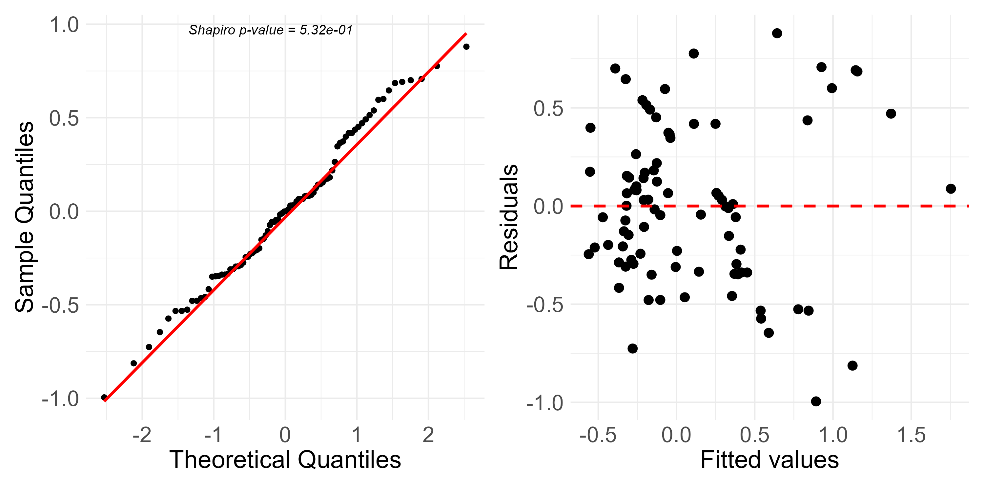


**Figure S7.** Residual analysis for the linear mixed effect models: *lm(response variable ~ temperature+ temperature : TP+ temperature : TP : B/P production ratio* with the response variables (a)phytoplankton DCA1 axis (b) zooplankton DCA1 axis and (c) fish DCA1 axis. On the left are Q-Q plots of the residuals from the linear models. The red line represents the reference line for a normal distribution. On the right the residuals from the linear models are plotted against the fitted values. The red dashed line at zero indicates no residual deviation. TP stands for total phosphorus and B/P production ratio for benthic/ whole lake primary production ratio.

Supplementary tables

**Table S1.** Meta table of all the lakes included in the analyses of this study. The 10 Swedish trend lakes are in bold and their rows highlighted. Under the column ‘Data’, phyto refers to lakes with data on phytoplankton biovolume, zoo for zooplankton biomass and fish for fish CPUE (catch-per-unit-effort) biomass. The presented values for surface water temperature, TP, TN and absorbance are the mean values of all samples in July and/ or August from 2004 – 2023. The presented values for air temperature are the mean values for the growth season (May – September) from 2004 – 2023.

| **EU ID** | **Lake name** | **Latitude (SWEREF 99 TM)** | **Longitude (SWEREF 99 TM)** | **Area (ha)** | **Mean depth (m)** | **Maximum depth (m)** | **Surface water temperature (°C)** | **Air temperature (°C)** | **TP (μg/l)** | **TN (μg/l)** | **Absorbance (a420)** | **Data** |
| --- | --- | --- | --- | --- | --- | --- | --- | --- | --- | --- | --- | --- |
| SE615365-134524 | Havgårdssjön | 6150322 | 396176 | 53 | 2.9 | 5 | 21.057 | 15.262 | 62.871 | 1030.429 | 1.263 | phyto, fish |
| SE615375-137087 | Krageholmssjön | 6150719 | 421789 | 209 | 4.5 | 8 | 20.280 | 15.078 | 158.660 | 1414.000 | 1.824 | fish |
| SE617797-135339 | Krankesjön | 6174721 | 404038 | 339 | 0.7 | 3 | 21.057 | 15.677 | 33.300 | 1042.714 | 2.303 | phyto, fish |
| SE622410-135589 | Fåglasjön | 6220852 | 405999 | 60 | 0.9 | 5 | 19.614 | 14.884 | 74.014 | 1179.857 | 23.039 | phyto, fish |
| SE622803-144609 | Svinarydsjön | 6225833 | 496102 | 19 | 1.2 | 2 | 20.929 | 15.124 | 17.857 | 667.429 | 3.007 | phyto |
| SE623624-141149 | Bäen | 6233634 | 461425 | 65 | 3.1 | 8 | 20.083 | 14.576 | 15.917 | 558.667 | 9.279 | phyto, fish |
| SE624038-143063 | Örsjön | 6237996 | 480506 | 19 | 3.2 | 9 | 20.543 | 14.710 | 9.714 | 469.000 | 4.289 | phyto, fish |
| SE624178-134911 | Lärkesholmssjön | 6238441 | 399017 | 76 | 4.2 | 8 | 19.871 | 14.711 | 16.986 | 1083.571 | 13.579 | phyto, fish |
| SE624421-147234 | Sännen | 6242312 | 522150 | 110 | 3.6 | 13 | 21.129 | 14.688 | 11.357 | 439.429 | 1.579 | phyto |
| SE624486-141154 | Skäravattnet | 6242250 | 461374 | 33 | 3.0 | 6 | 21.143 | 14.697 | 26.329 | 506.429 | 7.131 | fish |
| SE625612-138546 | Svanshalssjön | 6253198 | 435177 | 10 | 4.3 | 10 | 20.586 | 14.540 | 16.414 | 462.000 | 6.710 | phyto, fish |
| SE625911-138823 | Liasjön | 6256219 | 437910 | 12 | 2.5 | 4 | 19.586 | 14.307 | 45.600 | 1001.857 | 41.131 | fish |
| **SE627443-149526** | **Brunnsjön** | **6272787** | **544705** | **10** | **5.3** | **13** | **21.521** | **14.425** | **11.164** | **776.786** | **14.128** | **phyto, zoo, fish** |
| **SE628606-133205** | **Stora Skärsjön** | **6282493** | **381448** | **32** | **4.0** | **12** | **20.636** | **14.788** | **7.343** | **258.643** | **1.783** | **phyto, zoo, fish** |
| SE629570-135470 | Rammsjön | 6292542 | 403889 | 34 | 1.0 | 2 | 19.971 | 14.062 | 34.086 | 644.571 | 24.006 | phyto, fish |
| SE630558-134327 | Svartesjön | 6302132 | 392431 | 3 | - | 5 | 19.057 | 14.128 | 18.529 | 710.429 | 28.190 | phyto |
| SE630605-144655 | Hinnasjön | 6303819 | 495647 | 26 | 1.4 | 3 | 20.214 | 14.081 | 12.786 | 484.429 | 5.368 | phyto, fish |
| SE631360-146750 | Storasjö | 6311612 | 516497 | 37 | 1.8 | 6 | 20.157 | 13.818 | 12.729 | 389.429 | 5.960 | phyto, fish |
| SE632231-136476 | Harasjön | 6319106 | 413711 | 56 | 2.5 | 10 | 19.014 | 13.946 | 22.743 | 638.143 | 20.276 | fish |
| SE632515-146675 | Hjärtsjön | 6323148 | 515611 | 128 | 3.5 | 6 | 20.567 | 13.604 | 4.500 | 279.333 | 1.082 | phyto, fish |
| **SE633025-142267** | **Fiolen** | **6327724** | **471494** | **156** | **3.9** | **10** | **19.807** | **13.849** | **11.179** | **445.714** | **2.155** | **phyto, zoo, fish** |
| SE633209-141991 | Gyslättasjön | 6329531 | 468714 | 32 | 2.8 | 10 | 20.760 | 13.851 | 15.100 | 490.600 | 7.571 | phyto, fish |
| SE633344-130068 | Skärsjön | 6329472 | 349541 | 301 | 10.2 | 22 | 20.171 | 14.747 | 8.129 | 282.429 | 1.171 | phyto, fish |
| SE633738-142203 | Stora Skärsjön | 6334843 | 470770 | 29 | 3.8 | 14 | 20.486 | 13.958 | 16.571 | 595.286 | 14.704 | phyto, fish |
| SE633790-147515 | Kållen | 6336066 | 524356 | 27.82 | 2.7 | 10 | 20.400 | 13.732 | 42.500 | 1256.667 | 17.569 | phyto |
| SE633989-140731 | Älgarydssjön | 6337178 | 456028 | 34 | 1.4 | 7 | 18.871 | 13.889 | 17.229 | 538.714 | 8.006 | phyto, fish |
| SE634447-144024 | Holmeshultasjön | 6342144 | 488886 | 64 | 5.0 | 17 | 19.843 | 13.880 | 9.457 | 439.000 | 3.835 | phyto, fish |
| SE635878-137392 | Hagasjön | 6355662 | 422434 | 12 | 4.1 | 10 | 19.529 | 13.884 | 8.814 | 397.429 | 5.131 | phyto, fish |
| SE637120-145525 | Tångerdasjön | 6369038 | 503572 | 20 | 1.4 | 3 | 19.443 | 13.894 | 33.357 | 894.429 | 3.757 | phyto |
| SE637121-151366 | Tängersjö | 6369742 | 561955 | 11 | 3.1 | 9 | 21.314 | 14.495 | 7.171 | 521.571 | 2.566 | phyto, fish |
| SE638085-138862 | Mossjön | 6377893 | 436864 | 49 | 2.1 | 5 | 17.057 | 13.314 | 24.900 | 572.571 | 16.796 | phyto, fish |
| SE638317-138010 | Stengårdshultasjön | 6380110 | 428321 | 489 | 7.1 | 27 | 18.740 | 13.751 | 9.320 | 410.400 | 8.722 | phyto, fish |
| SE638665-129243 | Lilla Öresjön | 6382547 | 340666 | 61 | 4.1 | 17 | 20.014 | 14.456 | 9.229 | 360.000 | 4.566 | phyto |
| SE638725-146677 | Fjärasjö | 6385218 | 514896 | 35 | 4.3 | 13 | 19.714 | 13.708 | 7.186 | 398.000 | 2.572 | phyto |
| SE639047-149701 | Hökesjön | 6388795 | 545084 | 55 | 7.4 | 22 | 20.800 | 14.463 | 3.967 | 280.833 | 0.806 | phyto |
| SE640364-129240 | Stora Härsjön | 6399525 | 340434 | 257 | 14.1 | 42 | 17.737 | 14.616 | 4.600 | 313.500 | 2.602 | phyto, fish |
| SE640609-148673 | Skärgölen | 6404286 | 534623 | 6 | - | 7 | 20.471 | 13.864 | 6.529 | 372.286 | 2.033 | phyto |
| SE641603-144848 | Försjön | 6413766 | 496273 | 163 | 5.7 | 21 | 19.300 | 13.548 | 5.343 | 371.286 | 3.362 | phyto, fish |
| SE642008-168013 | Horsan | 6420575 | 727788 | 56 | - | 1 | 19.633 | 14.914 | 8.183 | 918.167 | 0.883 | phyto |
| SE642122-148744 | Glimmingen | 6421980 | 533782 | 175 | 10.4 | 32 | 20.743 | 13.820 | 4.457 | 292.000 | 1.125 | phyto |
| **SE642489-151724** | **Allgjuttern** | **6423441** | **564895** | **17** | **11.6** | **38** | **20.536** | **14.430** | **3.850** | **285.571** | **2.145** | **phyto, zoo, fish** |
| SE642555-168553 | Bästeträsk | 6426108 | 733122 | 639 | - | 4 | 20.033 | 14.888 | 3.917 | 661.833 | 0.760 | phyto |
| SE643914-127698 | Härsvatten | 6434816 | 324602 | 18 | 5.7 | 26 | 19.050 | 14.060 | 5.350 | 280.500 | 1.543 | phyto |
| SE644180-127892 | Torrgårdsvattnet | 6437498 | 326509 | 43 | 9.7 | 29 | 20.300 | 14.138 | 2.833 | 272.000 | 0.844 | phyto |
| SE644463-139986 | St. Lummersjön | 6441770 | 447338 | 5 | - | 6 | 18.286 | 13.651 | 13.029 | 354.857 | 18.197 | phyto |
| SE644987-152393 | Öjsjön | 6448490 | 571284 | 229 | 10.0 | 25 | 20.900 | 14.559 | 4.929 | 319.857 | 1.585 | phyto |
| SE645289-128665 | Fräcksjön | 6448672 | 334101 | 28 | 4.1 | 15 | 20.220 | 14.433 | 8.920 | 367.600 | 5.959 | phyto, fish |
| SE646293-126302 | Granvattnet | 6458422 | 310367 | 18 | 1.6 | 3 | 19.850 | 14.265 | 25.367 | 502.667 | 4.275 | phyto, fish |
| SE647050-130644 | Alsjön | 6466507 | 353666 | 6 | 5.0 | 10 | 20.043 | 13.942 | 15.143 | 619.000 | 13.704 | phyto |
| SE649314-149514 | Geten | 6491396 | 541988 | 20 | 3.6 | 7 | 18.960 | 15.290 | 22.430 | 741.500 | 15.123 | phyto, fish |
| SE649570-139120 | Östen | 6491342 | 435889 | 671.21 | 0.6 | 1 | 19.900 | 14.999 | 41.750 | 545.000 | 4.640 | phyto |
| SE650061-142276 | Humsjön | 6497994 | 469555 | 25 | 4.5 | 13 | 19.771 | 14.407 | 8.857 | 341.000 | 1.678 | phyto, fish |
| SE651573-152481 | Skärgölen | 6514333 | 571374 | 17 | 7.1 | 13 | 20.810 | 14.711 | 6.043 | 339.857 | 2.335 | phyto, fish |
| SE652412-143738 | Långsjön | 6521667 | 483884 | 67 | 4.2 | 18 | 19.940 | 14.072 | 11.480 | 522.900 | 10.072 | phyto, fish |
| SE652707-159032 | Björken | 6526457 | 636722 | 137 | 12.5 | 23 | 21.114 | 14.408 | 7.114 | 385.143 | 2.566 | phyto, fish |
| **SE652902-125783** | **Rotehogstjärnen** | **6524402** | **304388** | **15** | **3.6** | **9** | **21.350** | **14.082** | **14.143** | **455.571** | **10.776** | **phyto, zoo, fish** |
| SE653737-125017 | Ejgdesjön | 6532654 | 296633 | 86 | 7.0 | 29 | 21.220 | 13.674 | 2.720 | 280.600 | 2.607 | phyto, fish |
| SE654798-158954 | Skillötssjön | 6547830 | 634885 | 111.58 | 3.7 | 6.5 | 20.229 | 14.667 | 40.600 | 657.143 | 1.099 | phyto |
| SE655209-126937 | Stora Tresticklan | 6547594 | 315641 | 108 | - | 20 | 18.743 | 13.215 | 3.457 | 286.857 | 3.809 | phyto |
| SE655275-153234 | Älgsjön | 6551428 | 578455 | 32 | 3.3 | 7 | 19.200 | 14.721 | 37.260 | 923.800 | 12.526 | phyto. fish |
| **SE655587-158869** | **Stora Envättern** | **6555227** | **634745** | **38** | **5.4** | **11** | **21.264** | **14.425** | **6.771** | **428.929** | **3.036** | **phyto, zoo, fish** |
| SE655863-129783 | Västra Solsjön | 6554474 | 344002 | 184 | 12.3 | 40 | 19.400 | 13.809 | 2.086 | 225.143 | 1.441 | phyto, fish |
| SE656263-156963 | Djupa Holmsjön | 6561754 | 615611 | 20 | 8.5 | 24 | 20.671 | 14.337 | 6.971 | 481.429 | 7.145 | phyto |
| SE656419-164404 | Stensjön | 6564214 | 689977 | 39 | 9.1 | 21 | 20.490 | 14.666 | 5.960 | 339.300 | 4.163 | phyto, fish |
| SE656612-164132 | Årsjön | 6566110 | 687234 | 21 | 3.8 | 11 | 21.530 | 15.032 | 4.250 | 310.400 | 3.182 | phyto, fish |
| SE656895-163948 | Långsjön | 6568917 | 685361 | 32 | 4 | 6.55 | 20.557 | 14.546 | 49.829 | 742.000 | 1.796 | phyto |
| SE656984-164254 | Albysjön | 6569844 | 688409 | 70 | - | 12 | 20.643 | 14.712 | 25.157 | 585.429 | 1.796 | phyto |
| SE658086-130264 | Bysjön | 6576747 | 348540 | 123 | 6.2 | 12 | 19.900 | 13.748 | 9.386 | 280.714 | 2.178 | phyto, fish |
| SE659105-133982 | Överudssjön | 6587380 | 385573 | 224 | 2.7 | 6 | 19.914 | 14.274 | 47.143 | 673.714 | 3.092 | phyto, fish |
| SE660688-164478 | Tärnan | 6606900 | 690200 | 105 | 4.3 | 12 | 20.200 | 14.574 | 12.586 | 490.857 | 3.382 | phyto, fish |
| SE660749-161885 | Fysingen | 6607195 | 664270 | 490 | 2.0 | 5 | 19.643 | 14.958 | 27.314 | 651.571 | 1.783 | phyto |
| SE661521-130182 | Ulvsjön | 6611064 | 347304 | 52 | 9.2 | 31 | 19.371 | 13.062 | 6.286 | 267.000 | 3.460 | phyto, fish |
| SE662682-132860 | Örvattnet | 6622992 | 373926 | 75 | 9.2 | 36 | 18.420 | 12.850 | 4.940 | 216.400 | 1.778 | phyto, fish |
| SE663365-161779 | Edasjön | 6633334 | 662893 | 17 | 3.0 | 5 | 20.300 | 14.475 | 42.814 | 695.857 | 4.671 | phyto |
| **SE663532-148571** | **Övre Skärsjön** | **6633396** | **530845** | **169** | **6.1** | **32** | **18.729** | **13.418** | **7.329** | **376.643** | **8.260** | **phyto, zoo, fish** |
| SE663907-156927 | Ekholmssjön | 6638162 | 614324 | 57 | 2.9 | 6 | 19.600 | 14.409 | 25.100 | 546.786 | 3.743 | phyto |
| SE664197-149337 | Dagarn | 6640136 | 538420 | 176 | 4.7 | 14 | 19.929 | 14.116 | 5.429 | 341.286 | 2.651 | phyto, fish |
| SE664410-136192 | Översjön | 6640666 | 407015 | 38 | 11.4 | 36 | 19.114 | 13.323 | 5.700 | 235.571 | 2.901 | phyto, fish |
| SE664620-148590 | V. Skälsjön | 6644274 | 530902 | 43 | 6.6 | 19 | 19.525 | 12.905 | 2.575 | 169.500 | 0.933 | phyto, fish |
| SE665175-157559 | Siggeforasjön | 6650914 | 620487 | 72 | 4.5 | 11 | 19.800 | 14.098 | 11.771 | 518.000 | 9.592 | phyto, fish |
| SE665654-149206 | Mäsen | 6654684 | 536934 | 43 | 9.6 | 22 | 19.514 | 14.097 | 8.543 | 380.714 | 2.730 | phyto |
| SE666268-142230 | Skifsen | 6659970 | 467134 | 35 | 2.6 | 10 | 18.700 | 13.259 | 8.840 | 336.800 | 7.258 | phyto, fish |
| SE667151-149602 | Hällsjön | 6669695 | 540709 | 20 | 5.3 | 18 | 19.686 | 13.685 | 5.443 | 408.000 | 3.632 | phyto |
| SE670275-146052 | Tryssjön | 6700486 | 504845 | 30 | 7.2 | 20 | 17.800 | 11.746 | 6.360 | 342.400 | 11.955 | phyto, fish |
| SE672467-148031 | Spjutsjön | 6722638 | 524356 | 36 | 5.9 | 21 | 18.843 | 13.697 | 3.157 | 201.000 | 1.368 | phyto |
| SE672729-138082 | Gipsjön | 6724037 | 424887 | 67 | 4.9 | 14 | 17.571 | 11.956 | 12.457 | 384.286 | 12.875 | phyto, fish |
| SE674570-141911 | Rädsjön | 6742906 | 462930 | 58 | 8.8 | 29 | 18.370 | 12.286 | 3.520 | 179.300 | 1.709 | phyto, fish |
| SE677506-156174 | Gosjön | 6774007 | 605132 | 39 | 2.0 | 5 | 19.243 | 13.328 | 21.043 | 560.714 | 12.579 | phyto |
| SE680235-141799 | Bösjön | 6799512 | 461113 | 114 | 4.2 | 17 | 16.360 | 10.579 | 5.780 | 213.900 | 4.891 | phyto, fish |
| SE683337-133785 | Ö. Särnamannasjön | 6829523 | 380637 | 28 | 1.9 | 6 | 12.975 | 8.435 | 3.000 | 87.250 | 0.472 | phyto, fish |
| SE683421-133742 | N. Särnamannasjön | 6830358 | 380196 | 35 | 1.8 | 5 | 13.175 | 8.480 | 5.400 | 135.000 | 0.553 | phyto, fish |
| SE683582-154935 | Källsjön | 6834590 | 591996 | 24 | 7.1 | 17 | 18.370 | 11.876 | 10.950 | 479.500 | 16.823 | phyto, fish |
| **SE683673-154083** | **Stensjön** | **6835394** | **583469** | **59** | **4.3** | **9** | **19.064** | **11.826** | **5.571** | **224.429** | **5.576** | **phyto, zoo, fish** |
| SE690617-134197 | Övre Fjätsjön | 6902328 | 383850 | 87 | 4.3 | 15 | 14.130 | 8.517 | 8.490 | 205.900 | 3.279 | phyto, fish |
| SE691365-156127 | V. Rännöbodsjön | 6912536 | 602945 | 44 | 6.2 | 20 | 19.100 | 12.626 | 11.114 | 303.143 | 3.783 | phyto, fish |
| SE695220-143383 | Stor-Backsjön | 6949478 | 475083 | 208 | 2.3 | 6 | 16.779 | 10.988 | 13.100 | 413.000 | 7.622 | phyto, fish |
| SE698860-135948 | Tronntjärnarna | 6984927 | 400320 | 3 | - | 5 | 9.187 | 5.779 | 2.033 | 60.000 | 0.491 | phyto |
| SE698918-158665 | Valasjön | 6988354 | 627371 | 178 | 9.0 | 26 | 18.514 | 12.545 | 10.414 | 348.286 | 7.375 | phyto, fish |
| SE704955-159090 | Hällvattnet | 7048754 | 630861 | 689 | 13.5 | 47 | 17.883 | 11.562 | 4.486 | 267.429 | 6.151 | phyto, fish |
| SE706083-132287 | Stor-Björsjön | 7056652 | 362825 | 45 | 4.7 | 15 | 13.244 | 9.393 | 3.821 | 169.429 | 3.987 | phyto, fish |
| SE706672-167201 | Svartvattnet | 7066942 | 711727 | 5 | - | 18 | 17.400 | 11.476 | 18.671 | 391.429 | 13.546 | phyto, fish |
| SE707669-170020 | Lill-Bursjön | 7077264 | 739783 | 10 | 1.4 | 3 | 18.384 | 12.170 | 13.286 | 533.143 | 8.724 | phyto, fish |
| SE708512-152086 | Degervattnet | 7083426 | 560401 | 166 | 6.7 | 18 | 18.280 | 11.704 | 4.557 | 246.286 | 3.414 | phyto, fish |
| **SE708619-162132** | **Remmarsjön** | **7085765** | **660807** | **131** | **5.3** | **14** | **18.208** | **11.684** | **8.975** | **288.333** | **7.614** | **phyto, zoo, fish** |
| SE709218-169710 | Sidensjön | 7092712 | 736488 | 9 | 2.6 | 4 | 17.969 | 12.097 | 28.029 | 580.286 | 8.835 | phyto, fish |
| SE711365-171748 | Täftesträsket | 7114434 | 756591 | 242 | 4.3 | 19 | 17.583 | 11.809 | 10.900 | 364.667 | 5.296 | phyto, fish |
| SE713131-144608 | Dunnervattnet | 7128648 | 485073 | 299 | - | 34 | 15.999 | 10.585 | 3.114 | 154.143 | 3.138 | phyto |
| SE713180-153188 | Djuphåltjärnen | 7130226 | 570825 | 6 | 3.0 | 4 | 15.620 | 11.329 | 35.400 | 475.333 | 16.156 | phyto, fish |
| SE713404-172465 | Bjännsjön | 7134909 | 763500 | 48 | 1.7 | 4 | 17.631 | 11.638 | 11.714 | 336.857 | 7.191 | phyto, fish |
| SE716717-158596 | Stor-Arasjön | 7166268 | 624434 | 730 | 7.3 | 22 | 13.745 | 9.709 | 6.475 | 193.000 | 3.604 | phyto, fish |
| SE717734-173458 | Brännvattsträsket | 7178324 | 772878 | 68 | 2.3 | 9 | 17.914 | 11.955 | 14.786 | 470.857 | 10.941 | phyto |
| SE718150-168580 | Vitträsket | 7181862 | 724058 | 198 | 6.8 | 21 | 16.857 | 11.534 | 3.529 | 153.286 | 0.750 | phyto |
| SE718284-148654 | Mellan-Rissjön | 7180666 | 524858 | 149 | - | 31 | 14.491 | 9.741 | 5.071 | 175.714 | 5.112 | phyto. fish |
| SE718898-170673 | Finnforsbodträsket | 7189607 | 744887 | 69 | 1.8 | 4 | 16.786 | 10.955 | 20.714 | 490.571 | 7.401 | phyto, fish |
| SE721811-171800 | Nyträsket | 7218872 | 755782 | 135 | - | 10 | 15.786 | 11.269 | 15.000 | 485.000 | 11.098 | phyto, fish |
| SE723383-175441 | Ytterträsket | 7235054 | 791982 | 10 | 2.8 | 6 | 17.900 | 11.853 | 11.886 | 302.286 | 10.993 | phyto, fish |
| SE726381-152328 | Magasjön | 7262068 | 560548 | 410 | - | 36 | 12.464 | 9.109 | 2.980 | 132.600 | 1.612 | phyto, fish |
| SE728744-162653 | Vuolgamjaure | 7287010 | 663453 | 209 | 4.1 | 15 | 15.771 | 10.030 | 4.171 | 191.429 | 2.487 | phyto, fish |
| SE731799-151196 | Stor-Tjulträsket | 7316078 | 548538 | 525 | 21.2 | 38 | 14.620 | 7.795 | 1.940 | 95.800 | 1.179 | phyto |
| SE733110-182955 | Bergträsket | 7333268 | 865858 | 20 | - | 7 | 16.629 | 11.816 | 22.929 | 346.286 | 7.401 | phyto |
| SE741340-153576 | Njalakjaure | 7411751 | 571098 | 33 | 5.8 | 20 | 10.410 | 7.508 | 2.040 | 69.700 | 0.594 | phyto, fish |
| SE742829-183168 | Pahajärvi | 7430468 | 866731 | 129 | 3.9 | 14 | 16.486 | 10.725 | 19.486 | 297.429 | 2.158 | phyto, fish |
| SE744629-167999 | Jutsajaure | 7446496 | 714847 | 112 | 1.9 | 9 | 15.514 | 9.526 | 7.643 | 233.571 | 3.941 | phyto, fish |
| SE751252-175433 | Valkeajärvi | 7513674 | 788304 | 62 | - | 11 | 16.386 | 9.959 | 4.757 | 162.429 | 0.928 | phyto |
| **SE758208-161749** | **Abiskojaure** | **7581424** | **650600** | **282** | **12.8** | **35** | **10.275** | **5.860** | **3.125** | **80.000** | **0.679** | **phyto, zoo, fish** |

**Table S2.** Outputs of the linear mixed effect models: *lme(ln F/Z Ratio ~ temperature, random = ~ 1 + year | lake)* with different lag times for fish carbon biomass in comparison to zooplankton carbon biomass. ‘F/Z Ratio’ stands for fish/ zooplankton carbon biomass ratio.

*Significance codes: 0 ‘***’ 0.001 ‘**’ 0.01 ‘*’ 0.05 ‘.’ 0.1 ‘ ’ 1*

| **Lag time (years)** | **Explanatory Variable** | **Estimate** | **SE** | **t-value** | **p-value** | **N** |
| --- | --- | --- | --- | --- | --- | --- |
| 0 | (Intercept) | 11.216 | 0.860 | 13.049 | 0.000*** | 97 |
|  | Temperature | -0.177 | 0.064 | -2.777 | 0.007** |  |
| 1 | (Intercept) | 11.453 | 0.846 | 13.541 | 0.000*** | 87 |
|  | Temperature | -0.195 | 0.063 | -3.099 | 0.003** |  |
| 2 | (Intercept) | 11.708 | 0.887 | 13.197 | 0.000*** | 77 |
|  | Temperature | -0.209 | 0.065 | -3.201 | 0.002** |  |
| 3 | (Intercept) | 12.180 | 0.862 | 14.127 | 0.000*** | 67 |
|  | Temperature | -0.244 | 0.064 | -3.836 | 0.000*** |  |

**Table S3.** Outputs of the linear mixed effect models: *lme(ln F/P Ratio ~ temperature, random = ~ 1 + year | lake)* with different lag times for fish carbon biomass in comparison to zooplankton carbon biomass. ‘F/P Ratio’ stands for fish/ phytoplankton carbon biomass ratio.

*Significance codes: 0 ‘***’ 0.001 ‘**’ 0.01 ‘*’ 0.05 ‘.’ 0.1 ‘ ’ 1*

| **Lag time (years)** | **Explanatory Variable** | **Estimate** | **SE** | **t-value** | **p-value** | **N** |
| --- | --- | --- | --- | --- | --- | --- |
| 0 | (Intercept) | 14.037 | 0.547 | 25.672 | 0.000*** | 195 |
|  | Temperature | -0.069 | 0.039 | -1.786 | 0.076. |  |
| 1 | (Intercept) | 14.229 | 0.570 | 24.974 | 0.000*** | 185 |
|  | Temperature | -0.085 | 0.041 | -2.083 | 0.039* |  |
| 2 | (Intercept) | 13.580 | 0.592 | 22.932 | 0.000*** | 175 |
|  | Temperature | -0.036 | 0.042 | -0.857 | 0.393 |  |
| 3 | (Intercept) | 14.649 | 0.551 | 26.597 | 0.000*** | 165 |
|  | Temperature | -0.119 | 0.040 | -2.970 | 0.003** |  |

**Table S4.** Conversion factors from phytoplankton biovolume (mm^3^ L^-1^) to carbon mass (g) (Blomqvist et al., 1995).

| **Phytoplankton Family** | **Conversion Factor** |
| --- | --- |
|  |  |
| Chlorophyta | biovolume /1000 * 0.16 |
| Cyanobacteria | biovolume /1000 * 0.22 |
| Bacillariophyta | biovolume /1000 * 0.11 |
| Charophyta | biovolume /1000 * 0.11 |
| Chrysophyceae | biovolume /1000 * 0.11 |
| Cryptophyta | biovolume /1000 * 0.11 |
| Dinophyceae | biovolume /1000 * 0.11 |
| Haptophyta | biovolume /1000 * 0.11 |
| Synurophyceae | biovolume /1000 * 0.11 |
| Raphidophyceae | biovolume /1000 * 0.11 |
| Xanthophyceae | biovolume /1000 * 0.11 |
| Euglenophyceae | biovolume /1000 * 0.11 |
| Choanoflagellidea | biovolume /1000 * 0.11 |
| Övriga Växtplankton | biovolume /1000 * 0.11 |

**Table S5.** Edibility factors of phytoplankton families (Lepori & Capelli, 2020).

| **Phytoplankton Family** | **Edibility Factor** |
| --- | --- |
| Bacillariophyta | 0.5 |
| Charophyta | 0 |
| Chlorophyta | 1 |
| Chrysophyceae | 1 |
| Cryptophyta | 1 |
| Cyanobacteria | 0 |
| Dinophyceae | 0 |
| Haptophyta | 0 |
| Övriga Växtplankton | 0 |
| Synurophyceae | 0 |
| Raphidophyceae | 0 |
| Xanthophyceae | 0 |
| Euglenophyceae | 0 |
| Choanoflagellidea | 0 |

**Table S6.** Species-specific trophic positions estimated from a number of food items from different studies using a randomised resampling routine (rfishbase; Boettiger et al., 2012).

| **Common name** | **Latin name** | **Trophic Position** |
| --- | --- | --- |
|  |  |  |
|  |  |  |
| Common Bream | *Abramis brama* | 3.150 |
| Common Bleak | *Alburnus alburnus* | 2.700 |
| Alpine Bullhead | *Alpinocottus poecilopus* | 2.980 |
| Eel | *Anguilla anguilla* | 3.670 |
| Blue Bream | *Ballerus ballerus* | 3.210 |
| Silver Bream | *Blicca bjoerkna* | 3.090 |
| Crucian Carp | *Carassius carassius* | 3.110 |
| Baltic Herring | *Clupea harengus* | 3.300 |
| Spined Loach | *Cobitis taenia* | *No data available* |
| Whitefish | *Coregonus lavaretus* | 3.150 |
| European Bullhead | *Cottus gobio* | 3.280 |
| Grass Carp | *Ctenopharyngodon idella* | 2.000 |
| Carp sp | *Cyprinus carpio* | 3.050 |
| Pike | *Esox lucius* | 4.470 |
| Three-spined Stickleback | *Gasterosteus aculeatus* | 3.380 |
| Gudgeon | *Gobio gobio* | 3.130 |
| Ruff | *Gymnocephalus cernua* | 3.260 |
| Pumpkinseed | *Lepomis gibbosus* | 3.270 |
| Asp | *Leuciscus aspius* | 4.470 |
| Vendace | *Leuciscus idus* | 3.790 |
| Common Dace | *Leuciscus leuciscus* | 2.930 |
| Burbot | *Lota lota* | 4.050 |
| Fourhorn Sculpin | *Myoxocephalus quadricornis* | 3.850 |
| Round Goby | *Neogobius melanostomus* | 3.300 |
| Rainbow Trout | *Oncorhynchus mykiss* | 3.530 |
| Sockeye Salmon | *Oncorhynchus nerka* | 3.230 |
| European Smelt | *Osmerus eperlanus* | 3.460 |
| Perch | *Perca fluviatilis* | 3.660 |
| Common Minnow | *Phoxinus phoxinus* | 3.270 |
| European Flounder | *Platichthys flesus* | 3.300 |
| Nine-spined Stickleback | *Pungitius pungitius* | 3.350 |
| Roach | *Rutilus rutilus* | 2.870 |
| Brown Trout | *Salmo salar* | 3.860 |
| Arctic Char | *Salvelinus alpinus* | 3.460 |
| Brook Trout | *Salvelinus fontinalis* | 3.740 |
| Lake Trout | *Salvelinus namaycush* | 3.770 |
| Zander | *Sander lucioperca* | 4.040 |
| Rudd | *Scardinius erythrophthalmus* | 2.890 |
| Wels Catfish | *Silurus glanis* | 4.120 |
| European Sprat | *Sprattus sprattus* | *3.010* |
| Common Chub | *Squalius cephalus* | 3.610 |
| Grayling | *Thymallus thymallus* | 3.140 |
| Tench | *Tinca tinca* | 3.270 |
| Vimba Bream | *Vimba vimba* | 2.750 |

**Table S7.** Model outputs from the linear models *(DCA1 ~ temperature + temperature : TP + temperature : TP : B/P production ratio).* DCA1 stands for DCA axis 1, TP for total phosphorus and B/P production ratio for benthic/ whole lake primary production ratio. Both response variables (phytoplankton and fish DCA1) were asinh-transformed prior to analysis to accommodate zero or negative values.

*Significance codes: 0 ‘***’ 0.001 ‘**’ 0.01 ‘*’ 0.05 ‘.’ 0.1 ‘ ’ 1*

| **Response Variable** | **Explanatory Variable** | **Estimate** | **SE** | **t-value** | **p-value** | **R2** | **adjusted R2** | **N** |
| --- | --- | --- | --- | --- | --- | --- | --- | --- |
| asinh (phytoplankton DCA1) | (Intercept) | 1.098 | 0.308 | 3.569 | 0.001*** | 0.266 | 0.244 | 107 |
|  | Temperature | -0.049 | 0.025 | -1.987 | 0.050* |  |  |  |
|  | Temperature : TP | -0.004 | 0.001 | -5.125 | 0.000*** |  |  |  |
|  | Temperature : TP : B/P production ratio | 0.005 | 0.001 | 4.657 | 0.000*** |  |  |  |
| asinh (fish DCA1) | (Intercept) | 3.124 | 0.287 | 10.885 | 0.000*** | 0.601 | 0.587 | 88 |
|  | Temperature | -0.233 | 0.023 | -10.066 | 0.000*** |  |  |  |
|  | Temperature : TP | 0.000 | 0.001 | -0.578 | 0.565 |  |  |  |
|  | Temperature : TP : B/P production ratio | 0.000 | 0.001 | 0.482 | 0.631 |  |  |  |

**Table S8.** Model outputs from the linear models lm(phytoplankton edibility ~ poly(phytoplankton DCA1) and lm(fish trophic position ~ poly(fish DCA1). DCA1 refers to the first axis from the DCA ordinations.

*Significance codes: 0 ‘***’ 0.001 ‘**’ 0.01 ‘*’ 0.05 ‘.’ 0.1 ‘ ’ 1*

| **Response Variable** | **Explanatory Variable** | **Estimate** | **SE** | **t-value** | **p-value** | **R2** | **adjusted R2** | **N** |
| --- | --- | --- | --- | --- | --- | --- | --- | --- |
| Phytoplankton edibility | (Intercept) | 0.367 | 0.010 | 37.721 | 0.000*** | 0.615 | 0.608 | 107 |
|  | Poly(DCA1, 2)1 | 0.945 | 0.101 | 9.391 | 0.000*** |  |  |  |
|  | Poly(DCA1, 2)2 | -0.889 | 0.101 | -8.828 | 0.000*** |  |  |  |
| Fish trophic position | (Intercept) | 3.451 | 0.009 | 384.231 | 0.000*** | 0.779 | 0.773 | 88 |
|  | Poly(DCA1, 2)1 | 0.888 | 0.084 | 10.539 | 0.000*** |  |  |  |
|  | Poly(DCA1, 2)2 | -1.155 | 0.084 | -13.706 | 0.000*** |  |  |  |

**Table S9.** Output from the linear model *lm(log(F/P biomass ratio) ~ temperature + temperature : TP + temperature : TP : B/P production ratio)* with fish catch data using both benthic and pelagic nets. F/P Carbon Ratio stands for fish/ phytoplankton carbon biomass ratio, TP for total phosphorus and B/P production ratio for benthic/ whole lake primary production ratio.

*Significance codes: 0 ‘***’ 0.001 ‘**’ 0.01 ‘*’ 0.05 ‘.’ 0.1 ‘ ’ 1*

| **Response Variable** | **Explanatory Variable** | **Estimate** | **SE** | **t-value** | **p-value** | **R2** | **adjusted R2** | **N** |
| --- | --- | --- | --- | --- | --- | --- | --- | --- |
| ln F/P Carbon Ratio | (Intercept) | 16.152 | 0.511 | 31.598 | <2E-16*** | 0.698 | 0.618 | 81 |
|  | Temperature | -0.173 | 0.042 | -4.095 | 1.03E-4*** |  |  |  |
|  | Temperature : TP | -0.0156 | 0.002 | -7.945 | 1.3E-11*** |  |  |  |
|  | Temperature : TP : B/P production ratio | 0.0170 | 0.003 | 6.825 | 1.78E-9*** |  |  |  |

**Table S10.** Output from the linear mixed effects models run on the temporal dataset with the northernmost lake (Abiskojaure, SE758208-161749) removed: *lme(response variable ~ temperature, random = ~ 1 + year | lake)*. The response variables include fish catch-per-unit-effort biomass (Fish CPUE), zooplankton to fish biomass ratio (Z/P Ratio), fish to zooplankton biomass ratio (F/Z Ratio), and fish to phytoplankton biomass ratio (F/P Ratio).

*Significance codes: 0 ‘***’ 0.001 ‘**’ 0.01 ‘*’ 0.05 ‘.’ 0.1 ‘ ’ 1*

| **Response Variable** | **Explanatory Variable** | **Estimate** | **SE** | **t-value** | **p-value** | **R2m** | **R2c** | **N** |
| --- | --- | --- | --- | --- | --- | --- | --- | --- |
| ln Phytoplankton Biomass | (Intercept) | -14.766 | 0.463 | -31.860 | 0.0000*** | 0.047 | 0.698 | 170 |
|  | Temperature | 0.094 | 0.032 | 2.931 | 0.0039** |  |  |  |
| sqrt Zooplankton Biomass | (Intercept) | 0.008 | 0.004 | 2.169 | 0.0333* | 0.005 | 0.494 | 84 |
|  | Temperature | 0.000 | 0.000 | 0.568 | 0.572 |  |  |  |
| ln Fish CPUE | (Intercept) | -1.100 | 0.419 | -2.625 | 0.0095** | 0.006 | 0.848 | 180 |
|  | Temperature | 0.042 | 0.027 | 1.573 | 0.118 |  |  |  |
| ln ZP Ratio | (Intercept) | 5.854 | 0.734 | 7.973 | 0.0000*** | 0.076 | 0.125 | 81 |
|  | Temperature | -0.122 | 0.053 | -2.310 | 0.0238* |  |  |  |
| ln FZ Ratio | (Intercept) | 9.137 | 0.922 | 9.906 | 0.0000*** | 0.003 | 0.536 | 74 |
|  | Temperature | -0.026 | 0.065 | -0.404 | 0.688 |  |  |  |
| FP Ratio | (Intercept) | 13.741 | 0.652 | 21.072 | 0.0000*** | 0.014 | 0.544 | 168 |
|  | Temperature | -0.061 | 0.046 | -1.323 | 0.188 |  |  |  |

**Table S11.** The most used taxonomic resources used in identification of phytoplankton.

| \| Bourrelly, P. 1966. Les algues d'eau douce. Tome I. Les algues vertes. – Editions N. Boubée & Cie, Paris. 511 pp. \| \| --- \| \| Bourrelly, P. 1968. Les algues d'eau douce. Tome II. Les algues jaunes et brunes.– Editions N. Boubée & Cie, Paris. 438 pp. \| \| Bourrelly, P. 1970. Les algues d'eau douce. Tome III. Les algues bleues et rouges. – Editions N. Boubée & Cie, Paris. 512 pp. \| \| Bourrelly, P. 1988. Compléments les algues d'eau douce. Tome I. Les algues vertes. – Société nouvelles des éditions boubée, Paris. 182 pp. \| \| Cleve–Euler, A., 1968. Die Diatomeen von Schweden und Finnland. – Bibliotheca Phycologica, Band 5. – Verlag von J. Cramer, New York. 961 pp. \| \| Coesel, P.F.M. & Meesters, J. 2013. European flora of the desmid genera Staurastrum and Staurodesmus. – KNNV Publishing, Zeist, the Netherlands. 357 pp. \| \| Croasdale, H & Flint, E.A. 1986. Flora of New Zealand. Freshwater algae, Chlorophyta, Desmids, Volume I – V.R.Ward, Government Printer, Wellington, New Zealand. 160 pp. \| \| Croasdale, H & Flint, E.A. 1988. Flora of New Zealand. Freshwater algae, Chlorophyta, Desmids, Volume II – V.R.Ward, Government Printer, Wellington, New Zealand. 180 pp. \| \| Croasdale, H, Flint, E.A. & Racine M.M. 1994. Flora of New Zealand. Freshwater algae, Chlorophyta, Desmids, Volume III – V.R.Ward, Government Printer, Wellington, New Zealand. 302 pp. \| \| Hindak F.2001. Fotograficky Atlas – VEDA, Bratislava.127 pp. \| \| Huber-Pestalozzi, G. ed. Die Binnengewässer, Band XVI. Das Phytoplankton des Süsswassers Teil 1 - 8. – E. Schweizer-bart'sche Verlagsbuchhandlung, Stuttgart. \| \| John M, Whitton B.A & Brook A.J. ed. 2003. The freshwater algal flora of the British Isles. An identifcation guide to freshwater and terrestrial algae. – Cambridge, University Press. 702 pp. \| \| John M, Whitton B.A & Brook A.J. ed. 2011. The freshwater algal flora of the British Isles. An identifcation guide to freshwater and terrestrial algae. Second edition. – Cambridge, University Press. 878 pp. \| \| Komárek, J. & Zapomelova, E. 2007. Planktic morphospecies of the cyanobacteroial genus Joosten, A.M.T. 2006. Flora of the blue-green algae of the Netherlands. – KNNV Publishing, Utrecht, the Netherlands. 239 pp. \| \| Kristiansen J & Preisig H.R.2001. Bibliotheca Phycologica Band 110. Encyclopedia of Chrysophyte Genera. – J.Cramer in der Gebruder Borntraeger Verlagsbuchhandling, Berlin Stuttgart. 260 pp. \| \| Kristiansen J. 2002 The genus Mallomonas (Synurophyceae) – A taxonomic survey based on the ultrastructure of silica scales and bristles. – Opera Botanica Number 139, Copenhagen 218 pp. \| \| Lenzenweger R. 1996. Bibliotheca Phycologica Band 101. Desmidiaceenflora von Österreich Teil 1. – J.Cramer in der Gebrüder Borntraeger Verlagsbuchhandling, Berlin Stuttgart. 162 pp. \| \| Lenzenweger R. 1997. Bibliotheca Phycologica Band 102. Desmidiaceenflora von Österreich Teil 2. – J.Cramer in der Gebrüder Borntraeger Verlagsbuchhandling, Berlin Stuttgart. 216 pp. \| \| Lenzenweger R. 1999. Bibliotheca Phycologica Band 104. Desmidiaceenflora von Österreich Teil 3. – J.Cramer in der Gebrüder Borntraeger Verlagsbuchhandling, Berlin Stuttgart. 218 pp. \| \| Lenzenweger R. 2003. Bibliotheca Phycologica Band 111. Desmidiaceenflora von Österreich Teil 4. – J.Cramer in der Gebrüder Borntraeger Verlagsbuchhandling, Berlin Stuttgart. 87 pp. \| \| Round F.E, Crawford R.M. & Mann D.G.1990. The Diatoms, biology & morphology of the genera – Cambridge, University Press. 747 pp. \| \| Skuja,H., 1948. Taxonomie des Phytoplanktons einiger Seen in Uppland, Schweden. – Symb. Bot. Upsal. IX : 3. 399 pp. \| \| Skuja,H., 1956. Taxonomische und biologische Studien über das Phytoplankton schwedischer Binnengewässer. – Nova Acta Reg. Soc. Sci Upsal. Ser.IV, Vol.16, No 3. 404 pp. \| \| Skuja, H., 1964. Grundzüge der Algenflora und Algenvegetation der Fjeldgegenden um Abisko in Schwedisch-Lappland. – Nova Acta Reg. Soc. Sci. Upsal. Ser.IV, Vol.18, No 3. 465 pp. \| \| Süßwasserflora von Mitteleuropa, Band 1, 1/2, 2/1, 2/2, 2/3, 3, 4, 6, 9, 10, 14, 16, 19/1, 19/2, 19/3, 20 – Flera förlag och upplagor. \| \| Tikkanen, T. & Willén, T., 1992. Växtplanktonflora. – Naturvårdverket. \| \| Wehr J.D. & Sheath R.G. ed. 2003. Freshwater algae of North America. Ecology and classification. – Academic Press, Elsevier Science.918 pp. \| \| Wolowski,K. & Hindak, F. 2005. Atlas of Euglenophytes. – VEDA, Publishing House of the Slovak Academy of Sciences. 136 pp. \| |
| --- | --- | --- | --- | --- | --- | --- | --- | --- | --- | --- | --- | --- | --- | --- | --- | --- | --- | --- | --- | --- | --- | --- | --- | --- | --- | --- | --- | --- |

**Table S12.** The most used taxonomic resources used in identification of zooplankton.

| Bartoš, E., 1959. Fauna ČSR, Vířníci – Rotatoria. Praha. 15: 969 pp. |
| --- |
| Berzins´, B. 1966. Djurplankton, kompendium. – Limnologiska institutionen, Lund. |
| Bick, H. et al 1972. Die Binnengewässer, Band XXVI. Das Zooplankton der Binnengewässer Teil I. – |
| E. Schweizer-bart´sche Verlagsbuchhandling, Stuttgart. 294 pp. |
| Błędzki, L.A. and Rybak, J.I. 2016. Freshwater Crustacean Zooplankton of Europe, Cladocera & Copepoda (Calanoida, Cyclopoida), Key to species identification, with notes on ecology, distribution, methods and introduction to data analysis; – Springer. 923 pp. (På biolabservern) |
| Brohmer, P. 1964. Fauna von Deutschland. – Quelle & Meyer Verlag, Heidelberg. 594 pp. |
| Djurplankton – Kompendium. 1976. – Limnologiska institutionen, Uppsala. 65 pp. |
| Dussart, B.H. and Defaye, D. 2001. Introduction to the Copepoda (2nd edition, revised and enlarged). Guides to the Identification of the Microinvertebrates of the Continental Waters of the World 16 Coordinating editor: H.J.F. Dumont – Backhuys Publishers, Leiden The Netherlands. 344 pp. |
| Einsle, U. 1993. Crustacea: Copepoda: Calanoida und Cyclopoida. Süsswasserfauna von Mitteleuropa 8/4-1. – Gustav Fischer Verlag, Stuttgart. 208 pp. |
| Einsle, U. 1996. Copepoda: Cyclopoida, genera Cyclops, Megacyclops. Acanthocyclops. Guides to the Identification of the Microinvertebrates of the Continental Waters of the World 10 Coordinating editor: H.J.F. Dumont – SPB Academic Publishing, The Hague, The Netherlands. 82 pp. |
| Flössner, D. 2000. Die Haplopoda und Cladocera (ohne Bosminidae) Mitteleuropas . – Backhuys Publishers, Leiden. 429 pp. (På biolabservern) |
| Herbst, H.V. 1962. Blattfusskrebse. – Kosmos. Stuttgart. 130 pp. |
| Kiefer, F. 1960. Ruderfusskrebse (Copepoden) – Kosmos. Stuttgart. 97 pp. |
| Korovchinsky, N.M. 1992. Sididae & Holopediidae (Crustacea: Daphniiformes). Guides to the Identification of the Microinvertebrates of the Continental Waters of the World 3. – SPB Academic Publishing, The Hague, The Netherlands. 82 pp. |
| Koste, W. 1978. Rotatoria. Die Rädertiere Mitteleuropas 2. Auflage. – Gebrüder Borntraeger,Berlin, Stuttgart.I. Textbd. 1-673, II. Tafelbd. T 1-234. |
| Stuttgart.I. Textbd. 1-673, II. Tafelbd. T 1-234. |
| Lieder, U. 1996. Crustacea: Cladocera: Bosminidae. Süsswasserfauna von Mitteleuropa 8/2-3. – Spektrum Akademischer Verlag, Heidelberg. 80 pp. |
| Liljeborg, W. 1982. Cladocera Sueciæ, del I, II, III. – Almqvist & Wiksell International Stockholm, Sweden. 701 pp. |
| Nogrady T. and Segers H. 2002. Rotifera. Volume 6: Asplanchnidae, Gastropodidae, Lindiidae, Microcodidae, Synchaetidae, Trochosphaeridae and Filinia. Guides to the Identification of the Microinvertebrates of the Continental Waters of the World 18 Coordinating editor: H.J.F. Dumont – Backhuys Publishers, Leiden. The Netherlands. 221 pp. |
| Pontin. R.M. 1978. A key to British Freshwater Planktonic Rotifera. – Freshwater Biological Association, Scientific publication No 38. 178 pp. |
| Ranga Reddy Y. 1994. Copepoda, Calanoida, Diaptomidae: key to the genera Heliodiaptomus, Allodiaptomus, Neodiaptomus, Phyllodiaptomus, Eodiaptomus, Arctodiaptomus and Sinodiaptomus. Guides to the Identification of the Microinvertebrates of the Continental Waters of the World 5 Coordinating editor: H.J.F. Dumont – SPB Academic Publishing, The Hague, The Netherlands. 221 pp |
| Rylow, W.M. Die Binnengewässer, Band XV. Das Zooplankton der Binnengewässer – E. Schweizer-bart’sche Verlagsbuchhandling, Stuttgart. 171 pp. |
| Røen, U.I. 1995. Krebsdyr V. Gællefødder og Karpelus. Danmarks Fauna 85. – Dansk Naturhistorisk Forening, København. 358 pp. |
| Srámek-Husék R, Straskroba, M. & Brtek, J. 1962. Fauna CSSR, Lupenonozci, (Branchiopoda). – Nakladatelstvi, Praha. 470 pp. |
| Ueda H. and Reid J. W. 2003. Copepoda: Cyclopoida, genera Mesocyclops and Thermocyclops. Guides to the Identification of the Microinvertebrates of the Continental Waters of the World 20 Coordinating editor: H.J.F. Dumont – Backhuys Publishers Leiden. The Netherlands. 318 pp. |
| Wallace R.L., Snell T.W., Ricci C. and Nogrady T. 2006. Rotifera: Volume 1: Biology, Ecology and Systematics (2nd edition). Guides to the Identification of the Microinvertebrates of the Continental Waters of the World 23 Coordinating editor: H.J.F. Dumont – Backhuys Publishers, Leiden, The Netherlands. 299 pp. |

References

Bergström, A., Lau, D. C. P., Isles, P. D. F., Jonsson, A., & Creed, I. F. (2022). Biomass, community composition and N:P recycling ratios of zooplankton in northern high‐latitude lakes with contrasting levels of N deposition and dissolved organic carbon. *Freshwater Biology*, *67*(9), 1508–1520. https://doi.org/10.1111/fwb.13956

Blomqvist, P., Bell, R. T., Olofsson, H., Stensdotter, U., & Vrede, K. (1995). Plankton and water chemistry in Lake Njupfatet before and after liming. *Canadian Journal of Fisheries and Aquatic Sciences*, *52*(3), 551–565. https://doi.org/10.1139/f95-056

Boettiger, C., Lang, D. T., & Wainwright, P. C. (2012). rfishbase: Exploring, manipulating and visualizing FishBase data from R. *Journal of Fish Biology*, *81*(6), 2030–2039. https://doi.org/10.1111/j.1095-8649.2012.03464.x

Jaspers, C., Nielsen, T. G., Carstensen, J., Hopcroft, R. R., & Møller, E. F. (2009). Metazooplankton distribution across the Southern Indian Ocean with emphasis on the role of Larvaceans. *Journal of Plankton Research*, *31*(5), 525–540. https://doi.org/10.1093/plankt/fbp002

Lepori, F., & Capelli, C. (2020). Seasonal variation in trophic structure and restoration effects in a deep perialpine lake (Lake Lugano, Switzerland and Italy). *Journal of Great Lakes Research*, *46*(4), 870–880. https://doi.org/10.1016/j.jglr.2019.12.008

Oksanen, J., Simpson, G. L., Blanchet, F. G., Kindt, R., Legendre, P., Minchin, P. R., O’Hara, R. B., Solymos, P., Stevens, M. H. H., Szoecs, E., Wagner, H., Barbour, M., Bedward, M., Bolker, B., Borcard, D., Carvalho, G., Chirico, M., De Caceres, M., Durand, S., … Weedon, J. (2022). *vegan: Community Ecology Package* (Version 2.6 4, p. 2.6-8) [Dataset]. https://doi.org/10.32614/CRAN.package.vegan

Vollenweider, R. A., & Kerekes, J. (1982). *Eutrophication of Waters*. 156.
